# Supplementary material for: Ensembles of Bidirectional Kinesin Cin8 Produce Additive Forces in Both Directions of Movement
Source: Biophys J. 2017 Nov 7;113(9):2055–67. doi: 10.1016/j.bpj.2017.09.006 (PMC5685778; doi:10.1016/j.bpj.2017.09.006)
Supplement: Document S1. Figs. S1–S6 and Table S1 [file mmc1.pdf]

Biophysical Journal, Volume *113*

Supplemental Information

Ensembles of Bidirectional Kinesin Cin8 Produce Additive Forces in Both Directions of Movement

Todd Fallesen, Johanna Roostalu, Christian Duellberg, Gunnar Pruessner, and Thomas Surrey

SUPPLEMENTAL TABLES AND FIGURES

Motor	% (-) Directed	% Not Moving	% (+) Directed	Total MTs
Kinesin-1	2.2	0.5	97.3	184.0
Cin8 5nM	53.0	16.7	30.3	66.0
Cin8 10nM	55.3	34.0	12.8	47.0
Cin8 50nM	0.0	13.6	86.4	110.0
Eg5	5.8	0.0	94.2	103.0

Table S1: Proportion of microtubules in plus and minus directions.
Total MTs refers to total number of microtubules observed for this calculation.

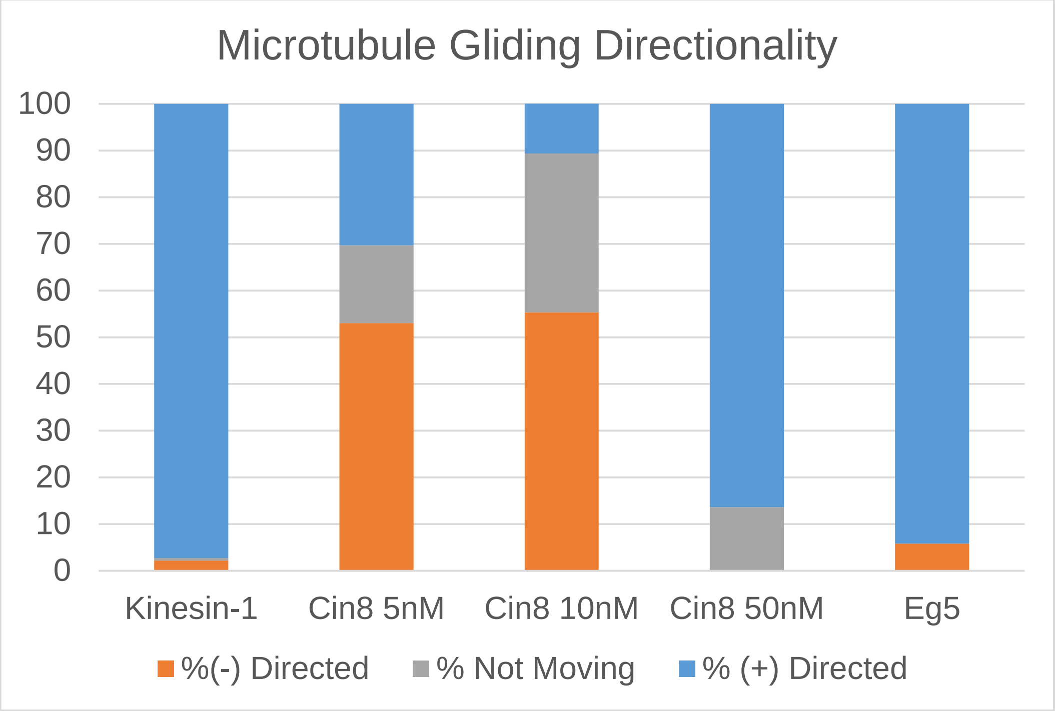

Figure S1. Microtubule polarity marking.
Gliding direction of polarity marked microtubules was observed for 250 nM kinesin-1, 5, 10 and 50 nM Cin8, and 200 nM Eg5. Approximately 98% of the microtubules gliding on kinesin-1 showed the bright polarity mark lagging, as expected for plus end directed motion. Therefore, only ~ 2% of the microtubules with a single polarity mark were incorrectly labelled.

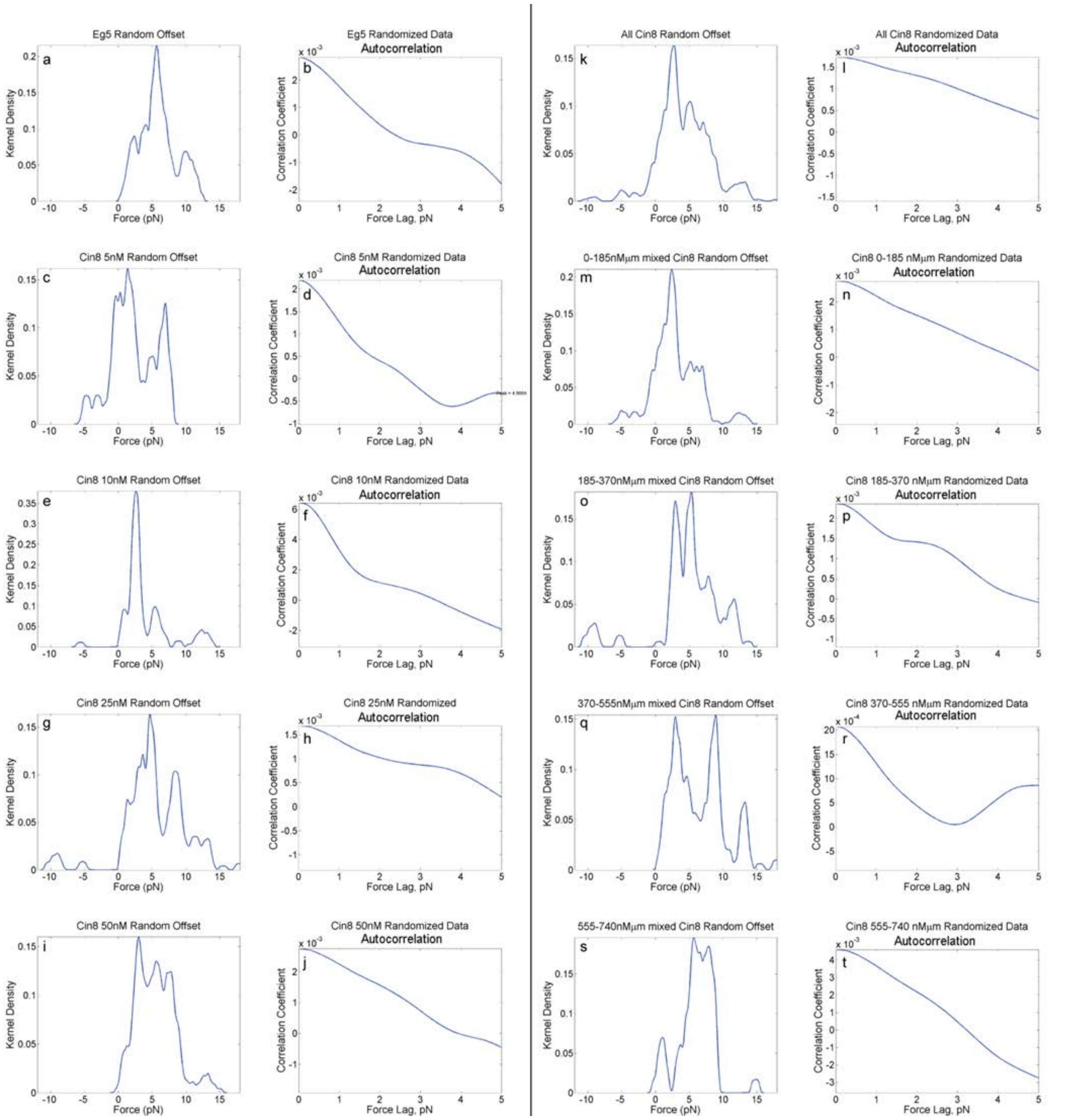

Figure S2. Analysis controls using randomized data

For control purposes, each data set was reanalyzed by kernel density estimation after adding a random value between 0-1.3 pN to each data point. No well-defined structure was seen in kernel density estimation after data randomization, and peaks were undefined in autocorrelations. Kernel density estimations (a, c, e, g, i, k, m, o, q, s) and the corresponding autocorrelation functions (b, d, f, h, j, l, n, p, r, t) are shown for the randomized data sets as indicated. Data presented in (k), (l) is for all Cin8 pause analysis data aggregated together.

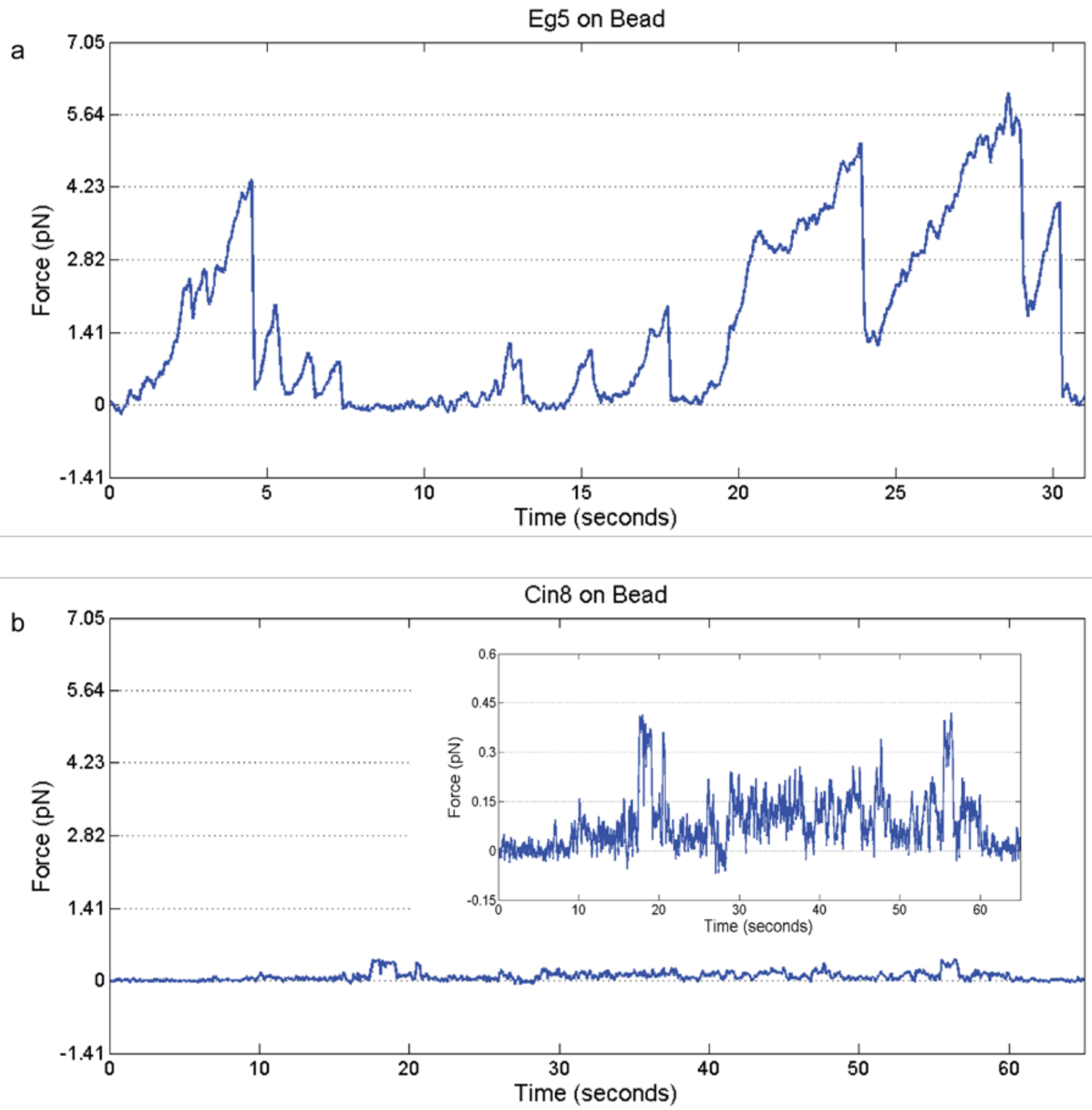

Figure S3. Eg5 and Cin8 on bead control

(a) Force generated against optical trap by Eg5-GFP molecules bound to beads, after beads are placed on surface immobilized microtubules. Trap stiffness is 0.061 pN/nm. Force generated is consistent with 1-4 molecules of Eg5. Eg5-GFP was bound to beads by anti-GFP antibodies. Dashed lines are spaced at 1.41 pN, the value found in the correlation in Figure 1e. ***(b)*** Force generated against optical trap by Cin8-GFP molecules bound via anti-GFP antibodies to beads, after beads are placed on microtubules fixed to surface. Trap Stiffness is 0.0061 pN/nm. Dashed lines are at 1.41 pN for comparison. Lack of force generation could be due to an intrinsic motile property of Cin8 through due to its bidirectionality, or may indicate that Cin8 is not compatible with bead attachment and optical trapping. ***(inset)*** Zoomed view of Cin8-GFP-microtubule interaction.

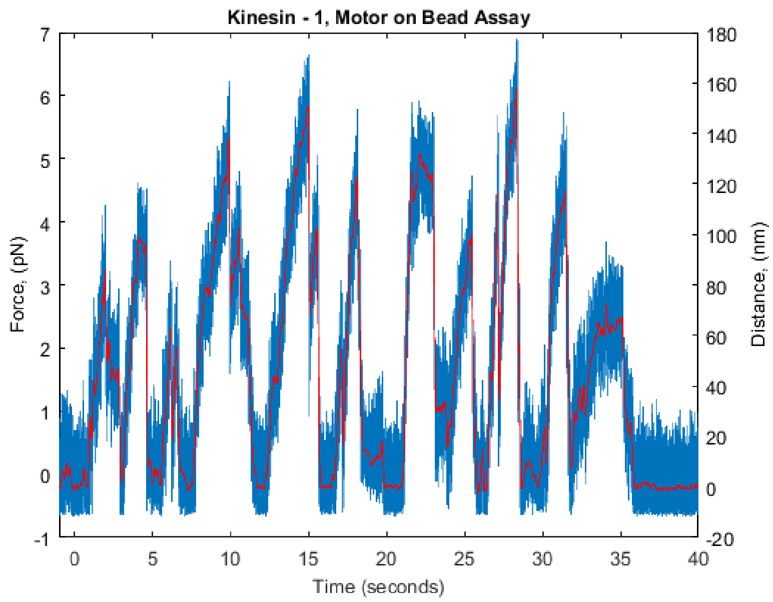

Figure S4. Kinesin-1 on bead control

Force generated by kinesin-1 molecules adsorbed to a caseine-coated carboxylated bead as the kinesin-1 walks along an immoblized microtubule transporting the bead out of the center of the optical trap. Trap stiffness was 0.0379 pN/nm.

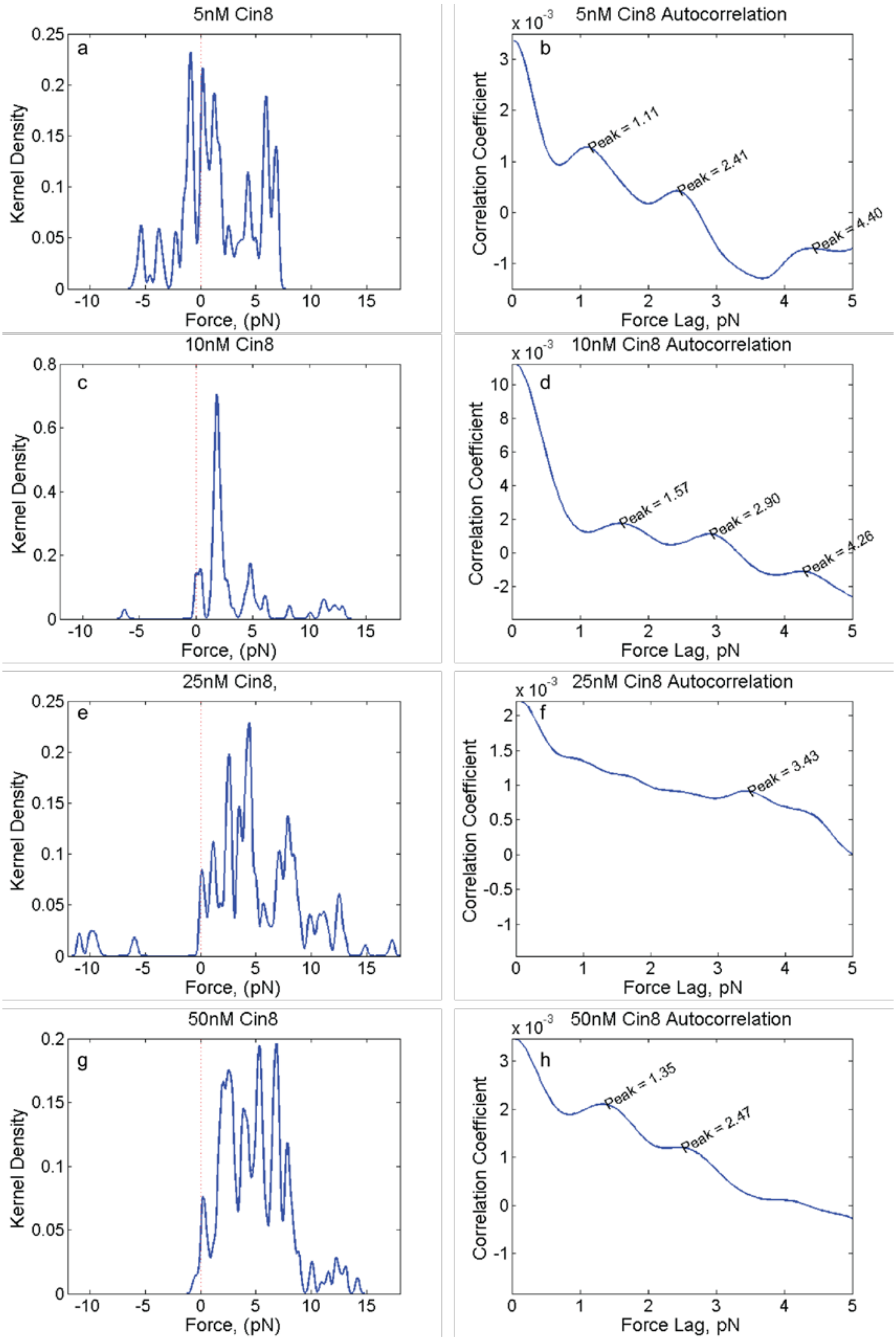

Figure S5. Autocorrelation analysis of Cin8 pause forces measured at different Cin8 concentrations

Kernel density estimation of Cin8 pause forces for experiments performed at different motor concentrations as indicated in panels a, c, e and g, while the autocorrelation functions for the respective Cin8 motor concentration is shown in panels b, d, f and h.

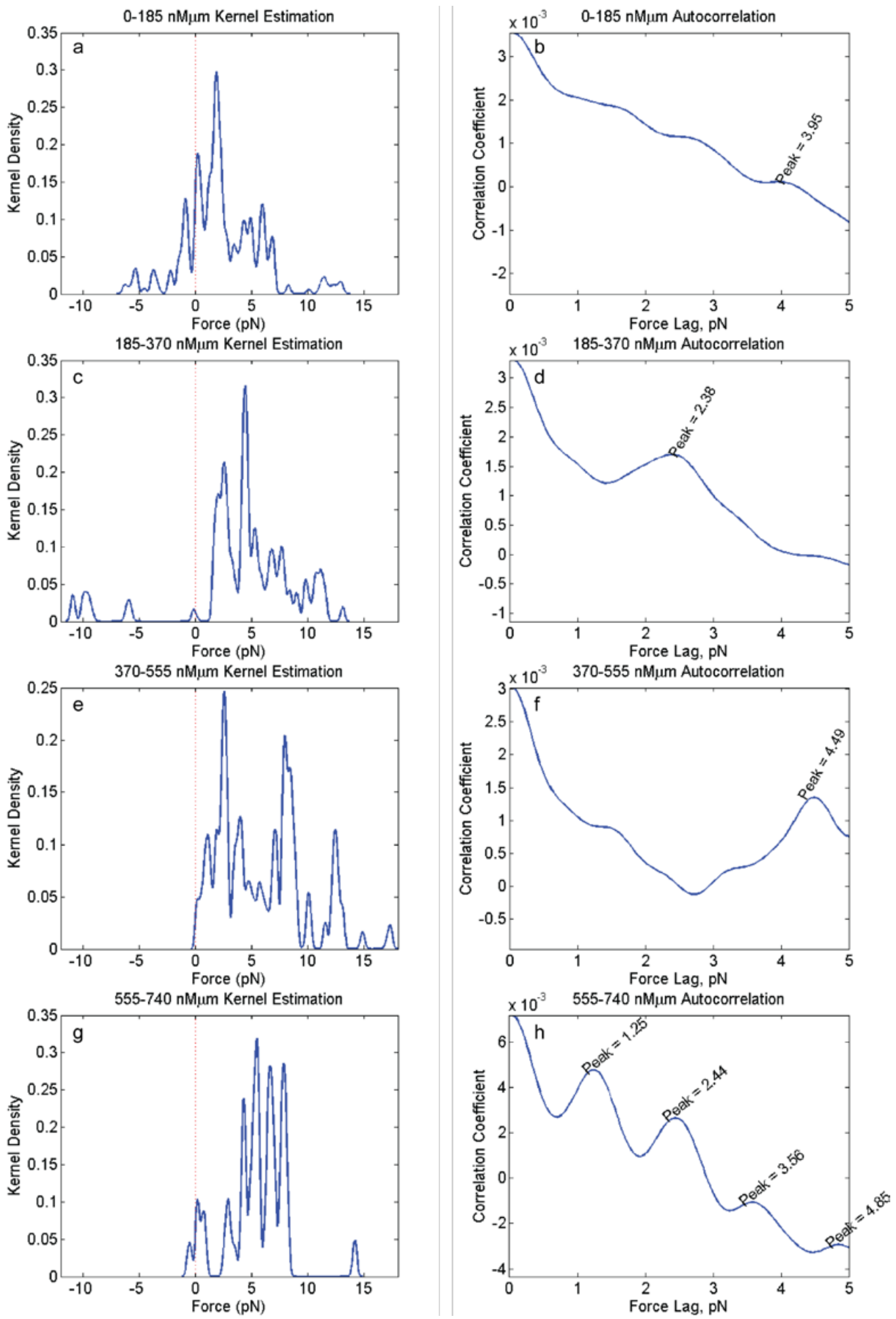

Figure S6. Autocorrelation analysis of Cin8 pause forces after recategorization

Kernel density estimation for all convoluted microtubule length – Cin8 concentration groups are shown in panels *a*, *c*, *e* and *g*. Autocorrelation functions for the respective panels are shown in *b*, *d*, *f* and *h*. Strong correlations are noted at higher nMμm categories, consistent with high motor number.
