# Supplementary material for: Ensembles of Bidirectional Kinesin Cin8 Produce Additive Forces in Both Directions of Movement
Source: Biophys J. 2017 Nov 7;113(9):2055–67. doi: 10.1016/j.bpj.2017.09.006 (PMC5685778; doi:10.1016/j.bpj.2017.09.006)
Supplement: Document S2. Article plus Supporting Material [file mmc2.pdf]

Biophysical*Journal*
Article

Ensembles of Bidirectional Kinesin Cin8 Produce Additive Forces in Both Directions of Movement

Todd Fallesen,[1] Johanna Roostalu,[1] Christian Duellberg,[1] Gunnar Pruessner,[2,*] and Thomas Surrey[1,*]
[1]The Francis Crick Institute and [2]Department of Mathematics, Imperial College London, London, United Kingdom

ABSTRACT Most kinesin motors move in only one direction along microtubules. Members of the kinesin-5 subfamily were initially described as unidirectional plus-end-directed motors and shown to produce piconewton forces. However, some fungal kinesin-5 motors are bidirectional. The force production of a bidirectional kinesin-5 has not yet been measured. Therefore, it remains unknown whether the mechanism of the unconventional minus-end-directed motility differs fundamentally from that of plus-end-directed stepping. Using force spectroscopy, we have measured here the forces that ensembles of purified budding yeast kinesin-5 Cin8 produce in microtubule gliding assays in both plus- and minus-end direction. Correlation analysis of pause forces demonstrated that individual Cin8 molecules produce additive forces in both directions of movement. In ensembles, Cin8 motors were able to produce single-motor forces up to a magnitude of ~1.5 pN. Hence, these properties appear to be conserved within the kinesin-5 subfamily. Force production was largely independent of the directionality of movement, indicating similarities between the motility mechanisms for both directions. These results provide constraints for the development of models for the bidirectional motility mechanism of fission yeast kinesin-5 and provide insight into the function of this mitotic motor.

INTRODUCTION

Members of the kinesin-5 family are essential for correct spindle assembly and spindle function in most eukaryotic organisms. They are important for separating centrosomes, connecting microtubules within the spindle, and exerting outward forces on mitotic half-spindles (1–5). Kinesin-5 motors are homotetramers with two N-terminal motor domains pointing in opposite directions and separated by a 60-nm-long stalk domain (6,7). This bipolar arrangement allows them to cross-link either parallel or antiparallel microtubules (8–10). Most kinesin-5 molecules studied so far, such as vertebrate Eg5 or *Drosophila* Klp61F are plus-end-directed motors; this is independent of whether single motors or ensembles of motors, either between antiparallel microtubules or on surface-immobilized microtubules, are observed (1,8–11).

In contrast, kinesin-5 family members from budding yeast (Cin8 and Kip1) and fission yeast (Cut7) have recently been discovered to be bidirectional (12–16). Similar to their metazoan counterparts, these fungal kinesin-5 motors are crucial for spindle assembly in early mitosis and also drive spindle elongation later in anaphase (17–19). In vitro experiments with purified proteins showed that single Cin8 and Cut7 molecules preferentially moved toward the minus ends of individual microtubules (12,14–16). This atypical directionality of movement for an N-terminal kinesin was reversed when these motors acted as part of a larger team, either when immobilized on a glass surface at high densities in microtubule gliding experiments or when sliding two antiparallel microtubules relative to each other (12,16). A recent theoretical model explained such motor-number-dependent directionality switching as a collective phenomenon resulting from an asymmetry of force-dependent motor properties in the presence of a diffusive motility component of a directional motor (20). The plus-end-directed motility mode of many Cin8 molecules is in agreement with their function in spindle-pole body separation and anaphase spindle elongation in yeast (17–19). The directionality of movement was also observed to be sensitive to the ionic strength (12,15), to the removal of positively charged parts of the motor (15,21), and to phosphorylation of the motor domain (22). All of these conditions are expected to lower the affinity of the motor for microtubule binding and hence also lower the number of motors participating in a team. It has

Submitted April 19, 2017, and accepted for publication September 6, 2017.
*Correspondence: g.pruessner@imperial.ac.uk or thomas.surrey@crick.ac.uk
Todd Fallesen's present address is Department of Life Sciences, Imperial College London, London, United Kingdom.
Christian Duellberg's present address is Institute of Science and Technology Austria, Klosterneuburg, Austria.
Editor: Stefan Diez.
https://doi.org/10.1016/j.bpj.2017.09.006
© 2017 Biophysical Society.
This is an open access article under the CC BY license (http://creativecommons.org/licenses/by/4.0/).

been hypothesized that directionality switching may allow the motors to position themselves close to microtubule minus ends early in prometaphase to accumulate the motors between spindle-pole bodies followed by plus-directed antiparallel microtubule sliding leading to spindle-pole body separation and later, in anaphase, to spindle elongation (12,23). Recent mathematical modeling of fission-yeast spindle assembly and spindle-pole separation supports this idea and suggests that bidirectionality of kinesin-5 motors is in fact essential for this process (24).

Force generation by kinesin-5 has so far been studied only for metazoan members of this family. Optical trapping experiments of full-length vertebrate Eg5 attached to micrometer-sized beads have revealed a maximum force production of up to ~1.5 pN per full-length molecule (25,26), which is about a quarter of the force that can be produced by kinesin-1 (27,28) or a truncated, dimeric version of Eg5 (29), possibly indicating a distinct influence of the C-terminal part of Eg5 on motor function. Furthermore, it was demonstrated recently that the force produced by teams of Eg5, which is the natural context in which they act on microtubule overlaps, depends linearly on motor number; in other words, forces of individual motors in a team add up (26).

Force production by fungal members of the kinesin-5 family has not yet been measured. Therefore, it is unknown whether the magnitude of the force production in the plus-end direction is a conserved property of kinesin-5 motors. Moreover, it is unknown whether the noncanonical minus-end-directed motility of fungal kinesin-5 can produce significant force, as is expected for a processively stepping motor, or whether motility in minus-end direction is potentially of a different origin, maybe involving diffusively bound states that would be expected to produce much lower forces. Finally, no measurements have been performed yet to test whether a hindering load can cause bidirectional kinesin-5 motors to stall like other motors or will induce a directionality switch.

To address these questions, we measure force production by bidirectional budding yeast kinesin-5 Cin8 in both directions. We used optical trapping of micrometer-sized beads attached to polarity-marked microtubules being propelled in gliding assays either by surface-immobilized full-length Cin8 or *Xenopus laevis* Eg5 motors. Using our optical trapping assay in conjunction with a newly developed correlation method to analyze collective motor stalls, we measured ensemble stall forces as multiples of single-molecule stall forces. Forces were measured for different Cin8 densities on the surface, leading to different microtubule gliding directionality and speeds (12). We find that Cin8 produces forces similar in magnitude to those observed for Eg5, in both plus- and minus-end direction, suggesting that minus-end-directed motility of Cin8 does not appear to result from a motility mechanism fundamentally different from that for plus-directed motility. Our analysis also indicates that, as is the case for Eg5, single-molecule forces of Cin8 add up in teams, albeit in plus- and minus-end-directed motility. Finally, the directionality of movement was insensitive to hindering loads up to the ensemble stall force.

MATERIALS AND METHODS

Protein preparations

Full-length budding yeast Cin8 with a C-terminal monomeric green fluorescent protein (GFP) tag (Cin8-mGFP) was prepared as described (12). Full-length *X. laevis* Eg5 with a C-terminal GFP tag was prepared as described (9), but imidazole was omitted from the final buffer. Full-length, non-fluorescently tagged *Drosophila melanogaster* kinesin-1 was prepared as described (30,31), with the exception that the elution buffer was not added as a gradient. For motor proteins, we state monomer concentrations throughout, unless indicated otherwise. Tubulin was purified from porcine brain, as described in (32). To generate biotinylated tubulin, Alexa647-tubulin, or NEM-tubulin, tubulin was covalently labeled with either EZ-Link NHS-LC-LC-biotin (21343, Life Technologies, Carlsbad, CA), Alexa Fluor 647-NHS (A-20006, Life Technologies), or N-(ethylmaleimide) (NEM) (Sigma-Aldrich, St. Louis, MO), respectively, essentially as described previously (33). All proteins were aliquoted in small aliquots, snap frozen, and stored in liquid nitrogen.

Motility assay with microtubules gliding over motor-coated surfaces

Preparation of polarity-marked biotinylated microtubules

Polarity-marked biotinylated microtubules were prepared in two steps (34). A dim mixture (DM), with 9 mg/mL tubulin consisting of 43% biotinylated tubulin, 43% unlabeled tubulin, and 14% Alexa647-tubulin in BRB80 (80 mM PIPES, 1 mM $MgCl_2$, and 1 mM EGTA (pH 6.8)), and a bright mixture (BM), with 8 mg/mL tubulin consisting of 40% NEM-tubulin (35), 32% Alexa-647 tubulin, and 28% biotinylated tubulin in BRB80, were prepared. The DM was mixed 1:1 with BRB80 supplemented with 1 mM GTP and 2 mM dithiothreitol (DTT) and incubated at 37°C for 45 min, adding 10% (v/v) 4 μM, 9.1% (v/v) 40 μM, and 8.3% (v/v) 400 μM taxol in BRB80 supplemented with 1 mM GTP and 2 mM DTT, after 15, 30, and 45 min, respectively (up to a final taxol concentration of 35 μM). After incubation, this DM microtubule suspension was further diluted 10-fold with BRB80 prewarmed to 37°C and supplemented with 1 mM GTP, 2 mM DTT, and 20 μM taxol. The BM was diluted 15-fold on ice with BRB80 supplemented with 1 mM GTP and 2 mM DTT. The diluted cold BM was added to the diluted DM mix at a ratio of 3:4 and polymerized for a further 15 min at 37°C before pelleting the microtubules at 95,000 $\times$ g at 37°C for 90 min through a cushion of 60% glycerol in BRB80 supplemented with 20 μM taxol. The polarity-marked microtubules were resuspended in BRB80 with 2 mM DTT and 20 μM taxol at room temperature and used within 10 h. Bright microtubule plus-segment labeling was verified by performing a gliding assay using 250 nM *Drosophila* kinesin-1 for surface immobilization (see below). Approximately half of the microtubules were polarity marked, and of those, the vast majority had bright plus-end segments in kinesin-1 gliding assays, as expected (Fig. S1; Table S1). Microtubules with ambiguous, multiple, or no polarity labels were not used for experimentation.

Preparation of streptavidin-coated micrometer-sized beads

Streptavidin-coated, 1.09 μm polystyrene beads (SVP-10-5, Spherotech, Lake Forest, IL; standard deviation of bead size, 0.160 μm) were diluted to 0.04% (w/v) in bead buffer (BB; phosphate-buffered saline (PBS) supplemented with 0.02% Brij-35) and were centrifuged for 5 min at 5000 $\times$ g at 4°C. After centrifugation, the beads were resuspended at the same density

in ice-cold BB and sonicated briefly (<10 s) in a 4°C water bath. After a second round of centrifugation, the beads were resuspended in ice-cold motility buffer (MB; 117 mM K-acetate, 72 mM PIPES, 150 mM KCl, 1 mM EGTA, 4 mM $MgCl_2$ (pH 6.85) supplemented immediately before use with 2 mM ATP, 4 mM DTT, 10 μM taxol, 1 mg/ml β-casein (Sigma, c6905), 0.04 mg/ml glucose oxidase (AMS Biotechnology, 22778.01), 0.02 mg/ml catalase (Sigma, C40), 40 mM D-glucose) and sonicated briefly in a 4°C water bath. The streptavidin beads were then diluted to a final concentration of 0.0001% (w/v) in MB at room temperature for force spectroscopy experiments on gliding microtubules.

Microtubule gliding assays

A reaction chamber was made by placing double-sided scotch tape (3M, Maplewood, MN) between a 22 × 22 and a 24 × 60 mm coverslip (thickness no. 1, Menzel-Gläser, Braunschweig, Germany). Glass coverslips were prepared as described in (12). MB was flowed into the chamber and allowed to incubate for 5 min at room temperature. A solution of Cin8-mGFP at 5–50 nM in MB was then flowed into the chamber and allowed to incubate for 5 min at room temperature. (Lower Cin8 concentrations could not be examined, because microtubules then frequently detached when the motors started displacing the microtubule-linked bead from the trap center.) Polarity-marked biotinylated microtubules in MB were then flowed into the chamber and allowed to incubate for 5 min at room temperature. For fluorescence imaging of microtubule gliding, the chamber was washed with MB to remove unbound microtubules before imaging. For force spectroscopy experiments on gliding microtubules, a dilute streptavidin bead sample (0.0001% w/v) in MB was flowed into the chamber. In all experiments, the chamber was sealed with vacuum grease (Beckman Coulter, Brea, CA) to prevent any flow in the sample due to drying out.

Microtubule gliding assays with kinesin-1 and Eg5 were performed as described for Cin8-mGFP above. However, the MB for kinesin-1 and Eg5-GFP was BRB80 supplemented with 2 mM ATP, 4 mM DTT, 10 μM taxol, 0.5 mg/mL β-casein, 0.04 mg/mL glucose oxidase, 0.02 mg/mL catalase, and 40 mM D-glucose immediately before use. Microtubules were allowed to bind for 10 min before washout. A concentration of 250 nM kinesin-1 or 200 nM Eg5-GFP was used. Kinesin-1 gliding assays were used only for imaging experiments, to verify that microtubule preparations were properly polarity marked.

To get a rough estimate of the number of Cin8 molecules interacting with a microtubule in a gliding assay, we calculate the total number of motors in the volume of a column bounded on both ends by a glass surface of 1 μm^2 and a height of 100 μm (which is the height of the experimental flow chamber) and assume that half the motors bind to each of the two glass surfaces, giving a surface density of ~375 tetrameric motors per 1 μm^2 at 50 nM (monomer concentration). Assuming a linear relationship between surface density and motor number interacting with the microtubule (31,36) and assuming that motors in a 50 nm wide area under a microtubule can reach the microtubule surface, we find that ~20 tetrameric motors per μm microtubule length are within reach of the microtubule at 50 nM (monomer concentration). This number likely reduces to ~2 motors/μm, because it has been previously reported that ~10% of motors are active in surface gliding assays (37).

Motility assay with motor-covered micrometer-sized beads on immobilized microtubules

Polarity-marked nonbiotinylated microtubules

Polarity-marked microtubules were prepared as above, substituting unlabeled tubulin for biotinylated tubulin, resulting in nonbiotinylated microtubules with brightly labeled plus-end segments.

Preparation of motor-coated beads

Carboxylated 1.1 μm beads (08226, Polysciences, Warrington, PA) were covalently coated with anti-GFP antibody (A-11120, Invitrogen, Carlsbad, CA) using the PolyLink Protein Coupling Kit (24350-1, Polysciences). The resulting anti-GFP beads were centrifuged and resuspended twice, as described for streptavidin beads. Beads that were to be used with kinesin-1 were not treated with anti-GFP, and instead were incubated in 1 mg/mL β-casein. Beads were then diluted 1:1 in Cin8, Eg5, or kinesin-1 MB (1 mg/mL β-casein was used in kinesin-1 and Eg5 MB here), as appropriate for the motor, and were allowed to absorb for 5 min on ice. Beads were then diluted 1:1 in a solution of GFP-tagged motors at 1–4 nM and allowed to bind the motors for 5 min on ice. Motor-coated beads were then further diluted 1:250 in the MB appropriate for the specific motor used. For experiments with Cin8-mGFP-coated beads, a range of Cin8-mGFP concentrations from 50 pM to 100 nM was explored, and absorption times ranging from 5 min to overnight were tried.

Motility assay with bead-immobilized motors

Glass surfaces were prepared and activated with glutaraldehyde, and sample chambers were prepared as described (38). Microtubules were flowed into the chamber and allowed to covalently react with the activated surface for 20 min before MB was flowed into the chamber to passivate the surface for 5 min at room temperature. Dilute motor-coated beads in MB were then flowed gently into the chamber and the sample was sealed with vacuum grease.

Optical trapping and fluorescence imaging

Optical trapping experiments were performed on a JPK Nanotracker 2 Optical Trap (JPK Industries, Berlin, Germany) using a Nikon Eclipse Ti-Inverted microscope (Nikon, Tokyo, Japan) as a base. This system was used earlier for biophysical force measurements in the range explored here (39). Fluorescence illumination was provided by an X-cite XLED1 (Excelitas, Waltham, MA) light source operating at 615–655 nm. The light source triggered the camera and was set to illuminate the sample for 100 ms at 5 Hz while beads were manipulated and for 100 ms at 0.2 Hz while Cin8 and Eg5 microtubules were tracked. Imaging of microtubules gliding on kinesin-1 was done with 100 ms illumination at 1 Hz due to the increased speed of kinesin-1 gliding. Trapping and epi-fluorescence imaging were both performed through the same Nikon CFI Plan Apochromat 60× WI, NA 1.20 objective. Images were collected on an Andor iXonEM 897 electron-multiplying charge-coupled-device camera. Trapping was done with a 3 W 1064 nm laser. Laser power was attenuated using a 1/4-wave plate and a circular polarizer to limit the amount of light that enters at the beginning of the optical path. We attenuated the beam to 1.7–5% of total power, so that the equivalent of 50–150 mW of power entered the optical path after attenuation. Bead deflections from the center of the trap were determined using back-focal-plane interferometry onto a quadrant photodiode. Before force spectroscopy commenced, each individual bead to be used was calibrated for stiffness using built-in software, utilizing a Lorentzian analysis of the power spectrum of the bead's Brownian motion (40). Correct calibration was verified by the manufacturer. Stiffness was varied due to experimental force needs, between 0.01 and 0.18 pN/nm, with a median value of 0.069 pN/nm for gliding assays. During force spectroscopy of beads coated with motors interacting with an immobilized microtubule, trap stiffness was varied between 0.006 and 0.06 pN/nm in an effort to find a range of stiffness where Cin8 movement would be observable. Force spectroscopy data were collected at 2 kHz.

Force spectroscopy experiments were performed by bringing a captured and calibrated streptavidin-coated bead into contact with a biotinylated, end-labeled microtubule (Fig. 1 *a*). An attempt was made to put the bead as close as possible to the "lagging" end of a moving microtubule, so that the majority of the motors would be pulling the bead out of the trap. Epifluorescence images were observed in real time and collected for time-lapse microscopy at 0.2–1 Hz to determine the directionality of the gliding microtubules. Beads that were coated with motor protein were captured and calibrated before being brought into contact with an

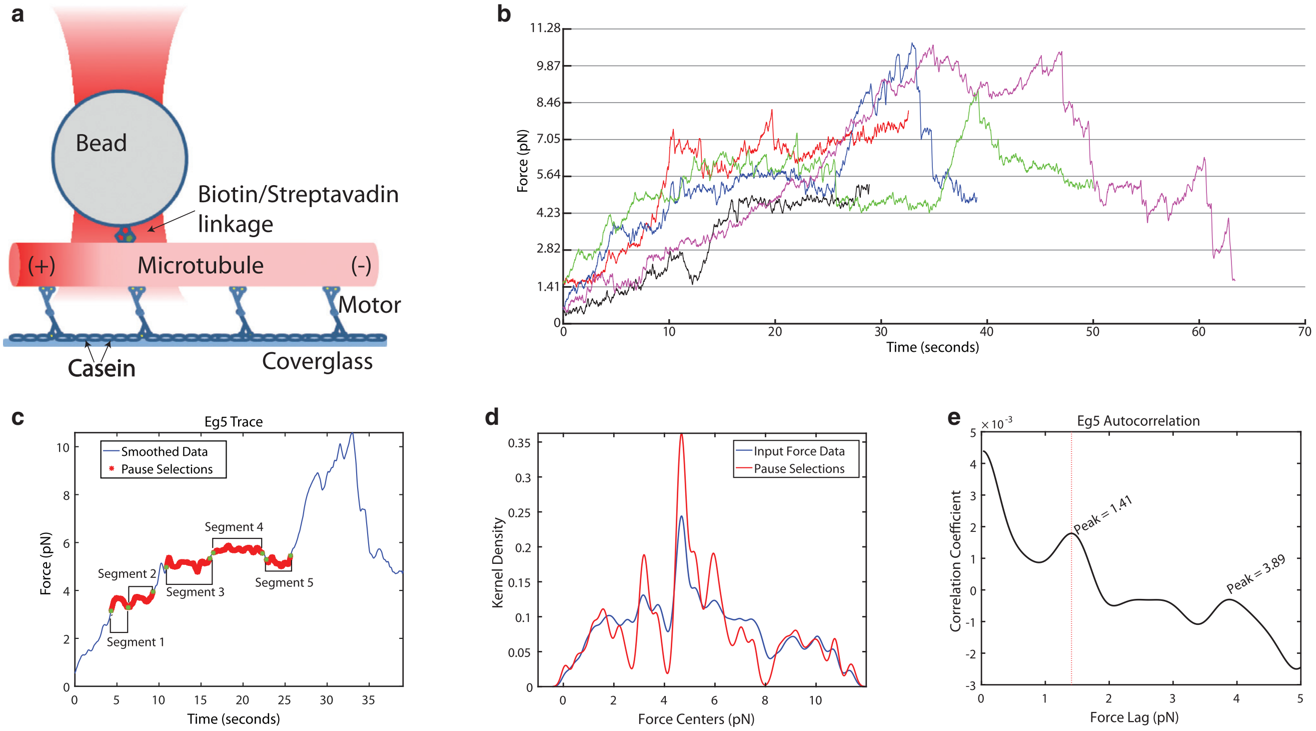

FIGURE 1 Correlation analysis of Eg5 pause forces. (*a*) Schematic diagram of the optical trapping assay. The tetrameric motor immobilized on a β-casein coated glass surface is propelling a microtubule. A micrometer-sized streptavidin-coated bead brought into contact with a biotinylated, end-labeled microtubule is trapped (Materials and Methods). (*b*) Example traces of five different Eg5-driven microtubules producing force against the optical trap. Data are displayed after a boxcar average over a 62.5 ms window. There were a total of 14 Eg5-driven microtubules tracked for a total of 432.4940 s (the total number of data points observed at 2 kHz was 864,988). Trap stiffness was on average 0.065 $\pm$ 0.006 pN/nm for these measurements. (*c*) Example trace showing selected pauses. Red regions are pauses selected using criteria described in the text. For this example, trap stiffness is 0.06 pN/nm. (*d*) Kernel-density estimation of Eg5 force distributions. The blue line represents all data before pause selection, the red line is all data after pause selection. The number of kernels is 500, and the bandwidth is 0.15 pN. Dashed lines are at a separation of 1.41 pN. The dashed line offset found by the minimization of distance from peaks to multiples of the stall force as extracted by the first peak from the autocorrelation function in (*e*). (*e*) Autocorrelation function of the Eg5 kernel-density estimation. The first peak is identified at 1.41 pN. To see this figure in color, go online.

immobilized microtubule. The resulting displacement of the bead from the center of the optical trap was recorded and used for force analysis (Fig. 1, *b* and *c*).

The quality of polarity-marked microtubules used for Cin8 experiments was determined by performing gliding experiments with kinesin-1. We performed a kinesin-1 gliding assay with each new set of microtubules to confirm proper polarity marking before commencing Cin8 experiments. Movies were recorded at 1 frame/s, and the number of microtubules with a single, unambiguous polarity mark was counted and their directionality noted. Typically, only ~2% of the tested microtubules had the polarity mark at the wrong end. Movies were taken during Cin8 and Eg5 force spectroscopy experiments at 5 frames/s, and microtubules were counted and their directionality noted. The directionality was determined for more microtubules than were used in force spectroscopy, as multiple microtubules could be moving in the background while we attached a bead to a microtubule for force spectroscopy (Fig. S1; Table S1).

Image analysis

Microtubule length and directionality were determined by video microscopy. Images were analyzed using the measurement tool in ImageJ to determine microtubule length, with an estimated error of ~0.4 μm. End labels of microtubules were present on all microtubules used in this study. Microtubules with their bright plus-end label observed during video playback to be at the end opposite the direction of travel were deemed "plus-end lagging" and were noted as microtubules driven by motors stepping in the plus-end direction. Likewise, microtubules with their plus-end label observed to be "plus-end leading," with the bright end label at the leading edge of the microtubule, were considered as microtubules driven by motors moving processively toward the minus-end direction.

Force analysis

All optical-trap force spectroscopy data were analyzed using in-lab-designed software written in MATLAB. All force data were boxcar averaged over 0.25 s before further analysis was performed. Pauses for each microtubule-bead gliding trial were algorithmically detected using the two criteria laid out in Eq. 1:

$$\begin{aligned} &F(t+DT)-F(t)<DF \\ &\text{Standard Deviation}(F(t)..F(t+DT))<\lambda. \end{aligned} \tag{1}$$

A given force at time "*t*" is represented by *F*(*t*). For the minimal allowed pause length (*DT*), the difference between the force at the end of the pause and the beginning of the pause must be less than *DF*. Further, we require that the standard deviation in the force spectroscopy data is $<\lambda$. In our data, we used a minimum pause length, *DT*, of 2 s, $DF = 0.2$ pN, and $\lambda = 0.15$ pN. *DT* was chosen to be conservatively high, a time in which Eg5 motors would be able to normally take multiple steps. Our value for

DF was chosen to allow a displacement of ~3 nm in our 2 s DT window at our median trap stiffness to account for fluctuations. The value for λ was chosen to be strict, to disallow any areas where the force may locally peak within a 2 s window, as motors become attached and disengaged. An example of pause detection is shown in Fig. 1 *c*.

We aggregated all of the force time segments that were determined to be "paused" (e.g., Fig. 1 *c*, *red segments*), from all force traces per condition to generate a distribution of forces at which the microtubule was "paused". Kernel density estimation was performed on this distribution in MATLAB using 500 normal distribution kernels having a bandwidth of 0.15 pN. This value was chosen based on automatic bandwidth estimation by MATLAB that suggested an optimal value of 0.1990 pN for a single underlying mode. With the expectation that there would be multiple peaks underlying the distribution, we conservatively reduced the bandwidth value to 0.15 pN (which is an order of magnitude smaller than the motor forces determined by our correlation analysis). We explored the parameter space of bandwidth and number of kernels, and curves generated with 50–1000 bins, and kernel bandwidths of 0.1–0.25 pN were comparable to our results, giving confidence in our parameters. Autocorrelation was performed on the kernel density estimation of the Eg5 and various Cin8 data sets to determine regularity of force-peak spacing. The autocorrelation function (41) (Eq. 2) quantifies the correlation of the data set $(a_1, a_2, \ldots, a_N)$ with a copy of itself displaced by i:

$$C_i = \frac{1}{N-i}\sum_{j=1}^{N-i}\left(a_j - \overline{a}_i\right)\left(a_{j+i} - \overline{a}'_i\right) = \left(\frac{1}{N-i}\sum_{j=1}^{N-i} a_j a_{j+i}\right) - \overline{a}_i\overline{a}'_i,$$

with

$$\overline{a}_i = \frac{1}{N-i}\sum_{j=1}^{N-i} a_j$$

and

$$\overline{a}'_i = \frac{1}{N-i}\sum_{j=1}^{N-i} a_{j+i}. \tag{2}$$

The normalization $N-i$ takes into account the decreasing overlap between the two copies as i increases and fewer data points become available. Removing this artifact is significant in this case, as the range of i considered is a significant fraction of N. Further, subtracting the product of the two window averages $\overline{a}_i$ and $\overline{a}'_i$, respectively, removes contributions to C_i from the mean value of a_i across the intervals $[1, N-i]$ and $[i+1, N]$, respectively.

The value at which the first peak in the autocorrelation occurs describes the interval between single-molecule stall forces, i.e., the single-molecule stall force.

Error analysis

Error of force estimation through autocorrelation

Error analysis on the autocorrelation data was done using a jackknife method, removing a subsample of the data and recalculating the statistical parameters (42). For our data, we removed the contribution of one microtubule trace per instance and calculated the position of the first peak in the autocorrelation data of the resultant kernel-density estimation. The data point locations in Fig. 5 (detailed in Table 2) are the average location of the first autocorrelation peak after each data subset removal. The error bars in Fig. 5 represent the standard error of that average (42).

Control of pause selection analysis

To reveal any underlying bias and to probe the sensitivity of the analysis, pause data for each data set was reanalyzed by randomization. For each data point in an observed pause, an independent, random offset value of 0–1.3 pN was added, and kernel-density estimation and autocorrelation analysis were done as described previously. No well-defined structure was seen in kernel-density estimation after data randomization, and peaks were undefined in autocorrelations (Fig. S2).

RESULTS

Force generation by motors on micrometer-sized beads

For control purposes, we first measured force production of kinesin-1 and Eg5. To this end, purified full-length kinesin-1 or full-length Eg5-GFP molecules were attached to micrometer-sized beads and individual beads were trapped using an optical trap, calibrated using power-spectrum analysis (40), and then brought into contact with prepolymerized and surface-immobilized taxol-stabilized microtubules (see Materials and Methods). We observed the expected motor-protein-driven processive displacement of the bead from the center of the optical trap for the two well-characterized motors with known motile properties. Forces of ~1.5 pN and up to 5–7 pN were generated for *X. laevis* kinesin-5 Eg5-GFP (Fig. S3 *a*) and *Drosophila* kinesin-1 (Fig. S4), respectively, in agreement with the range of previously measured single-molecule stall forces for these motors (25–28).

Beads coated with purified full-length Cin8-mGFP (Materials and Methods) were observed to bind to immobilized microtubules but, in contrast to control experiments, not to generate force that could displace the bead from the trap center, despite the experiments being conducted over a 100-fold trap-stiffness range (Fig. S3 *b*). Similar results were obtained for dimeric Cin8 constructs (data not shown). This might indicate either that Cin8 is not compatible with bead attachment and optical trapping or that its observed behavior is related to its bidirectionality. The reason is currently unknown, so to gain insight into the force generation by Cin8, we next measured the collective force production by several surface-immobilized Cin8 molecules in a microtubule gliding assay.

Force spectroscopy of collective Eg5 motor action

To determine the viability of a force spectroscopy assay of an ensemble of motors acting on a single microtubule, we again used the well-studied *X. laevis* kinesin-5 Eg5 as a control. To measure the collective force production, we modified the conventional gliding assay: 200 nM purified full-length Eg5-GFP was flowed into a flow chamber and allowed to attach to the glass surface, followed by the addition of taxol-stabilized, plus-end-labeled biotinylated

microtubules and followed by a dilute solution of streptavidin-coated polystyrene beads. Plus-end-directed gliding of end-labeled fluorescent microtubules over a surface coated with immobilized Eg5 was recorded using time-lapse fluorescence microscopy. Streptavidin-coated beads were optically trapped and brought into contact with a selected biotinylated microtubule (Fig. 1 *a*), whereupon the total force generated by the immobilized motors that interacted with this microtubule was recorded by measurement of bead displacement from the center of the optical trap (Fig. 1 *b*). Force calibration by power spectrum analysis was done for each bead before force spectroscopy commenced (Materials and Methods).

Force-spectroscopy traces of individual microtubule gliding events featured segments of varying speeds as well as segments of little microtubule motion (Fig. 1, *b* and *c*). In some cases, the microtubule was able to continue motion in its original direction of travel after a segment of constant force. We termed these segments, regardless of whether they were the terminally reached force or not, as "pauses." It is reasonable to assume that varying numbers of motors were in contact with the microtubule during a force recording and we attributed the pauses to the maximum force able to be reached by an ensemble of N motors. Given that it has been demonstrated that the forces produced by several Eg5 molecules acting as part of an ensemble are additive (26), we reasoned that it should be possible to extract the known maximum (or stall) force of single Eg5 molecules from the pause events in our ensemble data.

We first aggregated all force measurements from 14 force-time traces of Eg5-driven microtubule movement where the microtubule eventually reached a plateau, or "paused state," for a total of ~432.5 s (Table 1) (for representative force-time data, see Fig. 1 *b*) and analyzed the force data using kernel-density estimation, a continuous probability density estimation akin to histograms, generated by summing Gaussian bins (Fig. 1 *d*, *blue line*). This analysis showed the underlying structure of our force data, but did not allow discrimination of individual peaks of force representing pauses.

To discriminate for data in which the microtubule was "paused," we detected pauses algorithmically and aggregated the pauses (Materials and Methods). Detected pauses in an example force-time trace of a gliding microtubule are shown in Fig. 1 *c* (*red highlights*). Using the aggregated pause traces instead of the entire force-time trace data allowed for greater discrimination of the forces at which pauses occurred (Fig. 1 *d*, *red line*). For Eg5, we algorithmically detected the microtubule in a paused state for ~203.5 s (~47% of the time).

We then performed an autocorrelation analysis of the kernel density estimation of ensemble forces at pauses to estimate the peak-to-peak spacing (Fig. 1 *e*). The autocorrelation function shows a peak-to-peak spacing of 1.4 ± 0.2 pN, which is in good agreement with the previously reported value of 1.6 pN measured for single Eg5 molecules (25) and with single force estimates of 1.3 ± 0.6 pN from sequential force-induced cross-link ruptures of Eg5 molecules cross-linking microtubule pairs (26). This demonstrates that our autocorrelation-based analysis of the collective forces when motor-driven microtubules stall in a gliding assay can faithfully extract single molecule forces.

Force spectroscopy of collective Cin8 motor action

We next performed force spectroscopy on polarity-marked microtubules propelled by immobilized full-length Cin8 molecules. To measure the collective forces produced by Cin8 ensembles moving in either the plus or minus direction, we performed the gliding assay as described above for Eg5 with surface-immobilized Cin8-mGFP, using the motor at 5, 10, 25, or 50 nM.

At 50 nM motor concentration, all microtubules were observed to be propelled by ensembles of Cin8 motors moving and producing the net force in the plus-end direction (Fig. 2 *a*). A high quality of the microtubule polarity mark was confirmed in gliding experiments with kinesin-1 (Fig. S1; Table S1). After pause detection and kernel-density estimation, the resulting force distribution showed

TABLE 1 Experimental Parameters for Gliding Assay of Kinesin-5 Motors Against an Optical Trapping Force

Data Set	Minimal MT Length (μm)	Maximal MT Length (μm)	No. of Traces	Total Time (s)[a]	Total Time in Pause (s)[a]	% in Pause
Eg5	9.1	25.8	14	432.4940	203.5535	47.1
Cin8 5 nM	6.9	24.4	22	624.0950	488.7475	78.3
Cin8 10 nM	5.7	18.3	16	479.9005	244.7835	51.0
Cin8 25 nM	6.5	23.9	54	982.5305	435.0395	44.3
Cin8 50 nM	5.3	29.3	38	856.7845	433.0980	50.5
Cin8 negative	7.6	23.9	21	276.3525	175.6170	63.5
Cin8 positive	5.3	29.3	109	2666.9580	1499.9355	56.2
Cin8 0–185 nM μm	5.7	24.4	41	1218.005	866.4400	71.1
Cin8 185–370 nM μm	5.7	14.7	26	587.1465	270.6450	46.1
Cin8 370–555 nM μm	8.0	19.6	33	625.3105	293.3445	46.9
Cin8 555–740 nM μm	11.4	23.9	15	277.8710	112.8460	40.6

[a]Values are calculated from the total number of data points analyzed, acquired at 2 kHz.

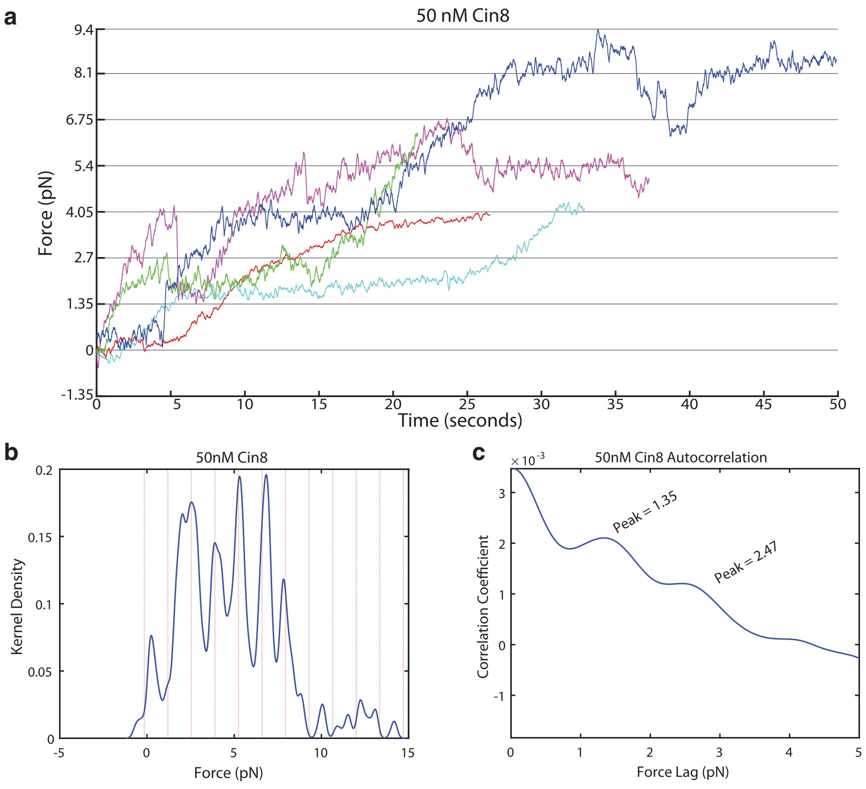

FIGURE 2 Cin8 pause forces at high motor densities. (*a*) Example traces of microtubules generating force against the optical trap, 50 nM Cin8 was surface immobilized for the gliding assay. The *Y* axis is marked by dotted lines at 1.35 pN intervals. There was a total of 34 microtubules observed for a total 856,7845 s at 2 kHz for 50 nM Cin8. (*b*) Kernel-density estimation of force production by 34 microtubules in a gliding assay, for the 50 nM Cin8 condition. The number of kernels is 500 and the bandwidth is 0.15 pN. The dotted lines are at a separation of 1.35 pN. The dashed line offset is found by minimization of the distance from peaks to multiples of the stall force as extracted by the first peak from the autocorrelation function in (*c*). (*c*) Autocorrelation function of the Cin8 kernel-density estimation. The first peak is identified at 1.35 pN. To see this figure in color, go online.

distinct peaks (Fig. 2 *b*), as observed above for Eg5. An autocorrelation analysis of the force distribution showed a spacing of 1.35 pN (Fig. 2 *c*), close to the value found for Eg5, indicating that the mechanism of plus-end-directed stepping is conserved for these two members of the kinesin-5 family. A jackknife error analysis (Materials and Methods) of this data set showed an average value of 1.3 ± 0.2 pN.

As the Cin8 concentration was lowered, microtubules exhibited motion in the minus-end direction (Figs. S1 and S5), as described earlier (12). At 5 nM Cin8, microtubules were observed to glide in both the plus and minus directions. There were a total of 22 microtubules analyzed at 5 nM for pause detection, of which 32% were gliding in the minus-end direction. (Note: The total fraction of microtubules gliding in the negative direction is not represented in our data, as we had a bias in choosing microtubules that were gliding in the minus-end direction and long enough to accurately place a bead on). Clearly, forces were observed to be produced in both directions of movement in this mixed data set (Fig. 3 *a*; Table 1). This was further confirmed by extraction of distinct peaks in the force distribution after pause detection and kernel-density estimation (Fig. 3 *b*). Autocorrelation analysis performed on the entire 5 nM Cin8 distribution spanning both plus- and minus-end-directed forces shows an average 1.11 pN stall force spacing (Fig. 3 *c*); this value may be reduced from the expected stall force as a consequence of a mixed directionality state. Autocorrelation analysis performed on only the plus-end-directed microtubules of the 5 nM Cin8 experiment showed a peak at 1.67 pN (Fig. 3 *d*) and autocorrelation analysis of only the minus-end-directed microtubules for the same condition shows a peak at 1.5 pN (Fig. 3 *e*), suggesting that mixed-directionality microtubules that were included in the analysis of the complete data set interfere with our method of analysis.

Indeed, when the data for all Cin8 gliding experiments at various concentrations were aggregated, pause-force analysis and kernel-density estimation of the force distribution (Fig. 4 *a*) and autocorrelation analysis showed no indication of a regular pause-force spacing (Fig. 4 *b*). This is consistent with the expectation that, especially at intermediate Cin8 concentrations, pauses might have been detected in a mixed-directionality regime of Cin8. This might lead to a broadening of the pause-force peaks, as some apparent "pauses" might not be a result of collective stalls of unidirectionally moving motors. This raised the question of whether our analysis of Cin8 force production in the plus versus the minus direction could be improved by better separating plus- and minus-directed motility from mixed-motility events.

Recategorization by number of active motors acting on the microtubule lattice

Our data for both Cin8 and Eg5, as well as previously reported data for Eg5 (26), support a hypothesis that the

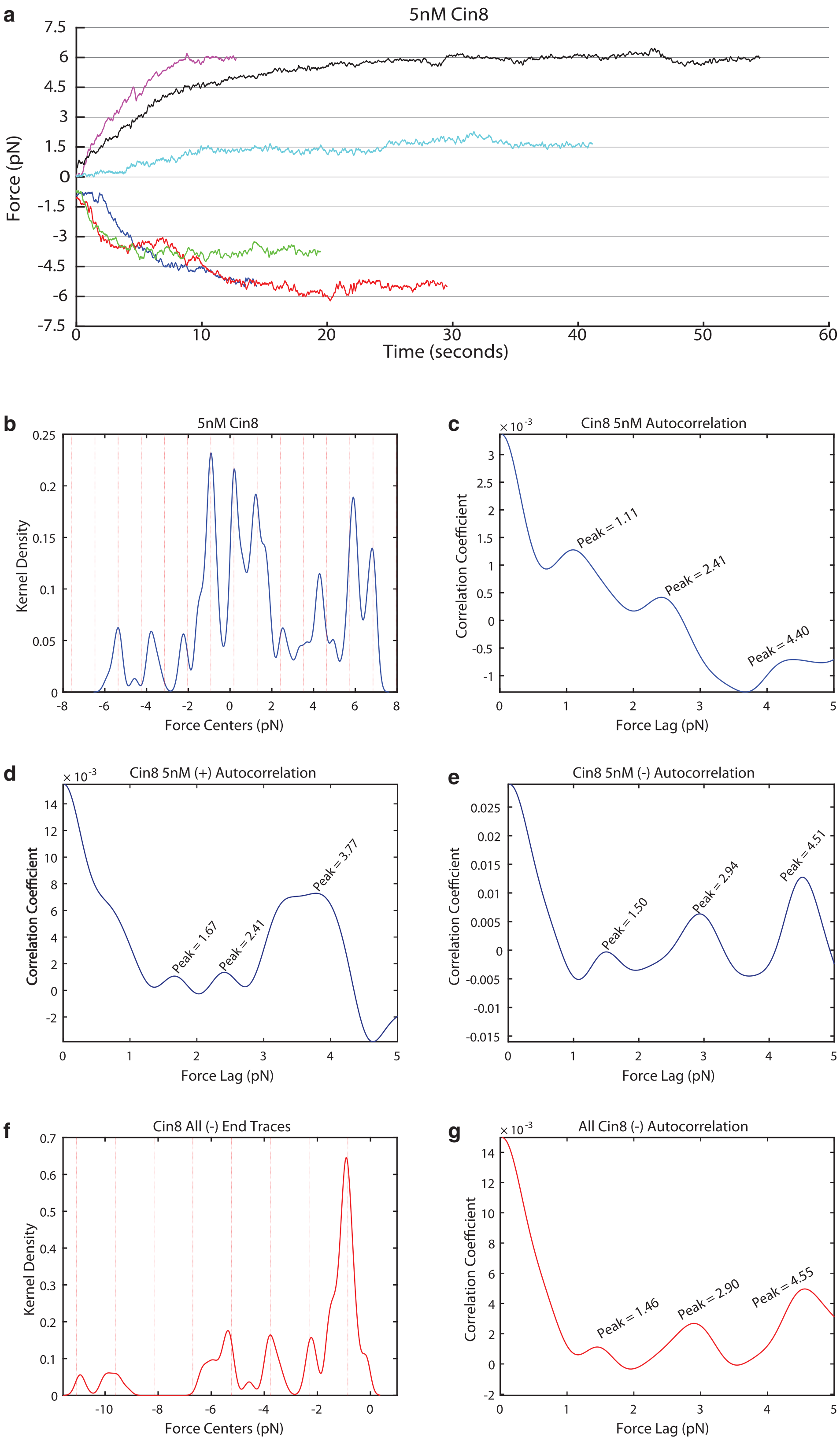

FIGURE 3 Cin8 pause forces at low motor densities. (*a*) Example traces of microtubules driven by plus- and minus-end-directed Cin8 motors, generating force against the optical trap, where 5 nM Cin8 was immobilized on the surface for the gliding assay. The *Y* axis is marked by dotted lines at 1.5 pN intervals for readability. There were a total of 22 microtubules tracked for a total of 624,1095 s at 2 kHz. (*b*) Kernel-density estimation of force production of all microtubules measured at 5 nM Cin8. (*c*) Corresponding autocorrelation plot of the kernel-density estimation in (*b*). The first peak is identified at 1.11 pN, giving the separation between dashed lines in (*b*). The dashed line offset is found by minimization of the distance from peaks to multiples of 1.11 pN. (*d* and *e*) Autocorrelation plots of the forces observed either in only the plus (*d*) or only the minus (*e*) direction at 5 nM Cin8. The first observed peaks are at 1.67 and 1.5 pN, respectively. (*f*) Kernel-density estimation of force production for all minus-end-directed microtubules combined that were detected in all gliding assays, with Cin8 concentrations ranging from 5–50 nM. (*g*) Autocorrelation function of the kernel-density estimation of the force distribution in (*f*). The first peak is identified at 1.46 pN, giving the separation between the dashed lines in (*f*) The dashed line offset is found by minimization of the distance from peaks to multiples of 1.46 pN. The number of kernels in the force distributions was always 500, and the bandwidth was 0.15 pN. To see this figure in color, go online.

number of motors interacting with a microtubule lattice directly relates to the total amount of force that can be generated by the gliding microtubule. Because microtubules in our assays showed a broad distribution of lengths (Table 1), we reasoned that regrouping the data into categories that reflect more directly the average numbers of force-generating motors per given microtubule would be beneficial. Therefore, we recategorized the data based upon the motor concentration used for immobilization multiplied by the microtubule length as an estimator of the average motor number acting upon the microtubule. Data were separated into four groups covering a range of 185 nM μm each (0–740 nM μm in total). Collective pause-force analysis was performed using pause detection, kernel-density estimation, and autocorrelation, as described before, now for the four new data groups (Fig. S6).

For the lowest motor number category, 0–185 nM μm, we note a large distribution of detected pause forces, similar to that in the previous 5 nM Cin8 category, with no clear autocorrelation when both plus- and minus-end-directed motility are grouped together (Fig. 4, *c* and *d*). The peak-to-peak separation became distinct for the highest motor number category, 555–740 nM μm (Fig. 4, *e* and *f*). In both the intermediate Cin8 concentration category of 25 nM (Fig. S5, *e* and *f*) and the intermediate motor-number categories (185–370 and 370–555 nM μm, Fig. S6, *c–f*) the autocorrelation function showed less distinct peaks. We hypothesize that at low motor number the spacing of pause events in the plus-end direction may be broadened by pauses caused by antagonistic motion of motors, i.e., some acting plus directed and some acting minus directed, such that peak-to-peak spacing representing single-molecule stall force cannot be extracted.

Stall force produced by single Cin8 in the plus direction

The recategorized data extracted from all the gliding experiments representing an expected high motor-number regime, the 555–740 nM μm Cin8-mGFP category (Fig. 4 *e*), showed repetition in the "pauses" of force every 1.25 $\pm$ 0.06 pN, as given by the sharp peak in the autocorrelation function (Fig. 4 *f*). The autocorrelation function further gives peaks at 2.44 and 3.56 pN, showing an approximate integer repetition of the 1.25 pN stall force. The repetition of the 1.25 pN spacing is overlaid on Fig. 4 *e* as dashed lines. This result is comparable to the peak observed at 1.35 pN in the autocorrelation function of the pause-force distribution for the condition with the highest-tested Cin8 concentration (Fig. 2, *b* and *c*). The values established here for plus-end-directed Cin8 are comparable to the established stall force for Eg5, and to the stall force of Eg5 determined by our method (25,26). The similarity of results given by analysis of Cin8 at 50 nM and the 555–740 nM μm recategorization suggest that the main parameter driving force production by the microtubule is the number of motors interacting with the microtubule, rather than the density of surface-immobilized motors.

Determination of the stall force of Cin8 in the minus direction

Minus-end-directed microtubule motion was only observed at low motor concentrations, and much fewer events were recorded than for plus-end-directed motion; thus, all minus-end-directed microtubule motion was grouped together (Fig. 3 *f*). At low motor concentrations, it is possible for microtubules to be plus- or minus-end-directed, or in a mixed state. To accurately measure the force of minus-end-directed Cin8, autocorrelation analysis was performed on only those microtubules observed to be driven by minus-end-directed Cin8, resulting in the detection of stall-force spacing of 1.46 $\pm$ 0.09 pN with subsequent peaks at 2.90 and 4.55 pN (Fig. 3 *g*). The similarity of this value to the stall force for Cin8 in the plus direction suggests that the stall force produced by Cin8 is essentially independent of the directionality of stepping. We note that to the best of our knowledge this is the first kinesin motor directly shown to generate force bidirectionally.

DISCUSSION

We have measured the increasing load generated by an ensemble of surface-immobilized Cin8 and Eg5 motors transporting a microtubule-attached microbead out of the center of an optical trap. Interpreting pauses of microtubule transport as ensemble stalls and using correlation analysis of stall-force histograms, we were able to show that ensemble stall forces appear to be quantized, suggesting that force production depends linearly on motor number and providing a convenient way to estimate the single-molecule stall force. Such a linear relationship between the ensemble force and the number of individual motors operating jointly in force production was recently demonstrated in a different manner for vertebrate Eg5 by directly comparing the ensemble force to individual motor dissociation events and measuring their dissociation force (26). Our correlation analysis of ensemble stall forces of Eg5 confirmed the additivity of single-molecule forces generated by this motor, and it offers an alternative method to measure and analyze force production of teams of motors.

Under our conditions, typically 10 or fewer molecular motors appeared to have contributed to the ensemble force generated by the moving microtubule against the optical trap. This range of the motor number agrees with a rough estimate of the expected number of surface-immobilized motors interacting with a microtubule, ~2–20 motors per 10 μm microtubule for 5–50 nM Cin8, respectively (Materials and Methods). We note that the forces generated are less than the maximum expected, consistent with a model in which not all motors that

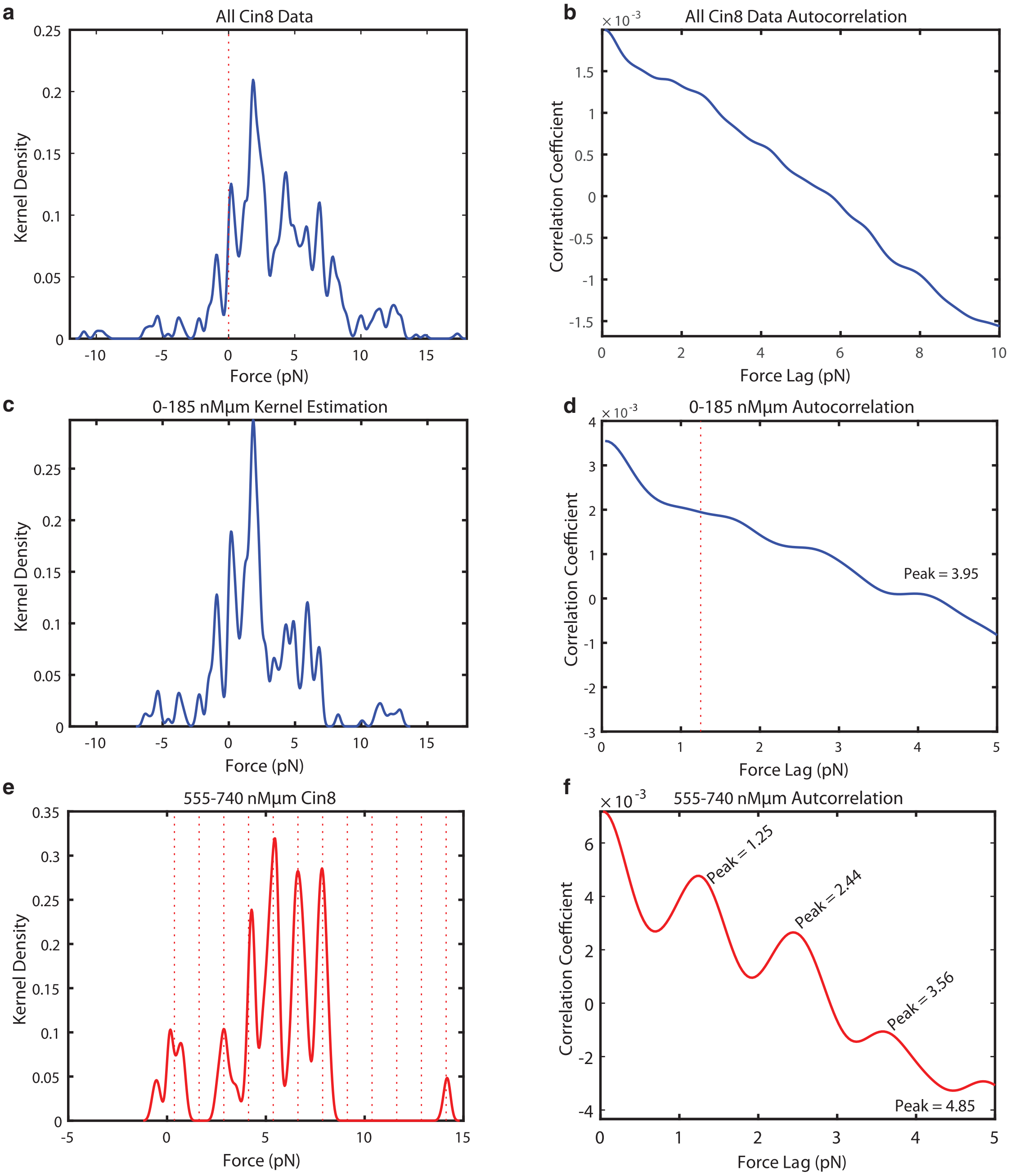

FIGURE 4 Analysis of subsets of pause-force data corresponding to different motor numbers. (*a*) Kernel-density estimation of detected pauses from all Cin8 experiments together. The dashed vertical line is at 0 pN. (*b*) Autocorrelation function of kernel-density estimation of all Cin8 data. No peaks were detected. (*c* and *d*) Kernel-density estimation (*c*) and its autocorrelation function (*d*) for the 0–185 nM μm Cin8 data. The peak is detected at 3.95 pN. (*e* and *f*) Kernel-density estimation (*e*) and its autocorrelation function (*f*) for the 555–740 nM μm Cin8 data. The first peak is identified at 1.25 pN. The dashed lines in (*e*) are at a separation of 1.25 pN. The dashed line offset is found by minimization of the distance from peaks to multiples of the stall force, as extracted by the first peak from the autocorrelation function. The number of kernels in the force distributions was always 500, and the bandwidth was 0.15 pN. The total number of microtubules observed, the total time tracked, and the time in pause are listed in Table 1. To see this figure in color, go online.

can interact with the microtubule lattice to generate force do so simultaneously, and some may be in a previously described diffusional state (12).

The main result of this study is that the bidirectional kinesin-5 Cin8 from budding yeast is able to produce piconewton forces in both directions of its movement with similar magnitude (Fig. 5; Table 2). This result indicates that like the more conventional plus-end-directed motility of this class of kinesins, minus-end-directed motility may also result from a more conventional motility mechanism instead of involving, for example, mostly diffusive motion that would be expected to produce much lower forces. A ratchet

model for minus-end motion, such as is observed for *Drosophila* kinesin-14, would not explain the generation of force; rather, it would predict a frictional braking against motion (43). Furthermore, Cin8 force production scales linearly with motor number, as recently shown for Eg5 (26), suggesting that additive force production is a conserved property of kinesin-5 motors, potentially reflecting a functional requirement for spindle assembly and elongation during cell division. Ensemble force production can, however, also be nonlinear for other members of the kinesin family, such as kinesin-1 (34,44). Our analysis also indicated that the magnitude of the single-molecule force produced by Cin8 and Eg5 is a conserved property of kinesin-5 motors (25,26), being distinct, for example, from the force produced by kinesin-1 (27,28,45).

We did not observe force-induced reversals of the direction of Cin8 motility while the load approached the ensemble stall force. Instead, microtubules almost always continued gliding in their original direction from the moment when the microtubule became attached to a trapped bead until it stalled. This indicates that the directionality of movement was determined when motility started in the absence of an external force. Directionality changes, as occasionally observed when imaging microtubule transport in the absence of load at intermediate Cin8 concentrations (12), may therefore result from variations of the number of motors interacting with the microtubule as it explores different surface areas of the sample. In force spectroscopy experiments, displacement of the microtubule by typically <500 nm when under observation, may be too small to allow the microtubule to enter an area of different local motor density.

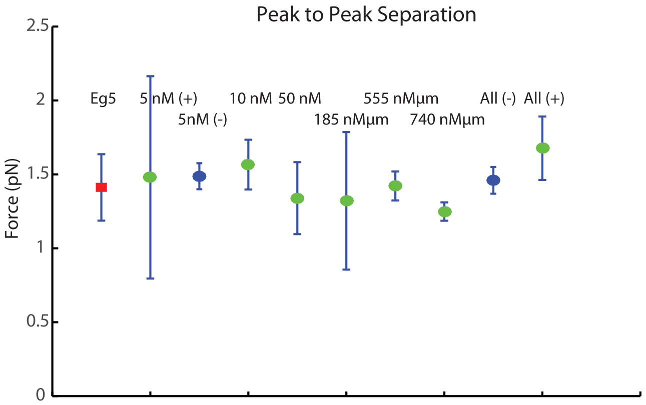

FIGURE 5 Summary of measured Cin8 forces. The average force for each Cin8 concentration condition (Eg5 as control (*red*)) and for each recategorized (MT length) *[Cin8] condition, as determined by the average position of the first peak in the autocorrelation of data during jackknife analysis. Error bars indicate the standard error of the force as determined by jackknife analysis. The data points for "5 nM (−)" and "All (−)," both in blue, represent force generated by microtubules moving in the minus-end direction, opposite the expected direction for N-terminal kinesin motors. Recategorized data sets are represented by the highest value in the data category (i.e., 0–185 nM μm is represented as 185 nM μm). To see this figure in color, go online.

TABLE 2 Average Force and Error as Determined by Jackknife Analysis of the Autocorrelation Peaks of Pause Detected Data for All Experimental Conditions

Experimental Condition	Average Value (pN)	Error (pN)
Eg5 (200nM)	1.4	0.2
Cin8 5 nM (+)	1.5	0.7
Cin8 5 nM (−)	1.49	0.09
Cin8 10 nM	1.6	0.2
Cin8 25 nM	ND	
Cin8 50 nM	1.3	0.2
Cin8 0−185 nM μm	1.3	0.5
Cin8 185−370 nM μm	ND	
Cin8 370−555 nM μm	1.4	0.1
Cin8 555−740 nM μm	1.25	0.06
Cin8 All	ND	
Cin8 All (−)	1.46	0.09
Cin8 All (+)	1.7	0.2

For details of the jackknife analysis (42), see Materials and Methods. ND, not determined.

We were unable to observe directly single-molecule force production of Cin8 attached to trapped micrometer-sized beads, although this was possible for Eg5 and kinesin-1. This may be due to a non-understood biochemical incompatibility of such a highly charged motor with functional bead surface attachment. Alternatively, this behavior may be related to the bidirectional nature of this motor potentially causing it to switch directionality or switch into a diffusive binding mode when steep load gradients are experienced upon single 8 nm steps, in contrast to the much smaller displacements of the microtubule when driven by a motor team (46). Nevertheless, thanks to the apparent cumulative property of forces produced by this motor, single-molecule forces in both directions could be determined indirectly from ensemble measurements.

Our observation of bidirectional force production by Cin8 puts constraints on models of the mechanism underlying the determination of the directionality of movement. Such models must account for significant force production in both directions of movement. For Cin8, it has been proposed that motor number, ionic strength, phosphorylation state, or distinct parts of the molecule that affect binding affinity are important determinants of the directionality of movement (12,15,21–23,47). In contrast, for the fission yeast ortholog Cut7, it has been suggested that crowding causing steric interference determines directionality (16). Interestingly, a fungal motor (KlpA from *Aspergillus nidulans*) from the generally minus-end-directed kinesin-14 subfamily, whose members, like those of the kinesin-5 subfamily (8), can also cross-link and slide antiparallel microtubules (48), has recently been reported to be able to behave also as a bidirectional motor (49). Whether all these bidirectional fungal motors use the same mechanism to switch from their canonical to their atypical directionality of movement is currently unknown and will require further study, most likely requiring a combination of mutational analysis and force spectroscopy.

SUPPORTING MATERIAL

Six figures and one table are available at http://www.biophysj.org/biophysj/supplemental/S0006-3495(17)31017-2.

AUTHOR CONTRIBUTIONS

T.F. performed the optical-trap experiments, analyzed data, and produced purified and labeled protein. T.F. and T.S. designed research with input from G.P. and J.R. J.R and C.D. provided purified protein. T.F. and T.S. wrote the manuscript with input from the other authors.

ACKNOWLEDGMENTS

The plasmid for full-length kinesin-1 was a gift from G. Holzwarth and J. Macosko with permission from J. Howard. We thank I. Lueke and N. I. Cade for technical assistance. G.P. thanks the Francis Crick Institute, and in particular the Surrey and Salbreux groups, for their hospitality during his sabbatical stay, as well as Imperial College London for making it possible.

This work was supported by the Francis Crick Institute, which receives its core funding from Cancer Research UK (FC001163), the United Kingdom Medical Research Council (FC001163), and the Wellcome Trust (FC001163), and by Imperial College London. J.R. was also supported by a Sir Henry Wellcome Postdoctoral Fellowship (100145/Z/12/Z) and T.S. by the European Research Council (Advanced Grant, project 323042).

REFERENCES

1. Sawin, K. E., K. LeGuellec, …, T. J. Mitchison. 1992. Mitotic spindle organization by a plus-end-directed microtubule motor. *Nature.* 359:540–543.
2. Tanenbaum, M. E., L. Macůrek, …, R. H. Medema. 2009. Kif15 cooperates with eg5 to promote bipolar spindle assembly. *Curr. Biol.* 19:1703–1711.
3. Sharp, D. J., G. C. Rogers, and J. M. Scholey. 2000. Microtubule motors in mitosis. *Nature.* 407:41–47.
4. Hildebrandt, E. R., L. Gheber, …, M. A. Hoyt. 2006. Homotetrameric form of Cin8p, a *Saccharomyces cerevisiae* kinesin-5 motor, is essential for its in vivo function. *J. Biol. Chem.* 281:26004–26013.
5. Hagan, I., and M. Yanagida. 1992. Kinesin-related cut7 protein associates with mitotic and meiotic spindles in fission yeast. *Nature.* 356:74–76.
6. Kashina, A. S., R. J. Baskin, …, J. M. Scholey. 1996. A bipolar kinesin. *Nature.* 379:270–272.
7. Scholey, J. E., S. Nithianantham, …, J. Al-Bassam. 2014. Structural basis for the assembly of the mitotic motor Kinesin-5 into bipolar tetramers. *eLife.* 3:e02217.
8. Kapitein, L. C., E. J. G. Peterman, …, C. F. Schmidt. 2005. The bipolar mitotic kinesin Eg5 moves on both microtubules that it crosslinks. *Nature.* 435:114–118.
9. Hentrich, C., and T. Surrey. 2010. Microtubule organization by the antagonistic mitotic motors kinesin-5 and kinesin-14. *J. Cell Biol.* 189:465–480.
10. van den Wildenberg, S. M., L. Tao, …, E. J. Peterman. 2008. The homotetrameric kinesin-5 KLP61F preferentially crosslinks microtubules into antiparallel orientations. *Curr. Biol.* 18:1860–1864.
11. Tao, L., A. Mogilner, …, J. M. Scholey. 2006. A homotetrameric kinesin-5, KLP61F, bundles microtubules and antagonizes Ncd in motility assays. *Curr. Biol.* 16:2293–2302.
12. Roostalu, J., C. Hentrich, …, T. Surrey. 2011. Directional switching of the kinesin Cin8 through motor coupling. *Science.* 332:94–99.
13. Fridman, V., A. Gerson-Gurwitz, …, L. Gheber. 2013. Kinesin-5 Kip1 is a bi-directional motor that stabilizes microtubules and tracks their plus-ends in vivo. *J. Cell Sci.* 126:4147–4159.
14. Edamatsu, M. 2014. Bidirectional motility of the fission yeast kinesin-5, Cut7. *Biochem. Biophys. Res. Commun.* 446:231–234.
15. Gerson-Gurwitz, A., C. Thiede, …, L. Gheber. 2011. Directionality of individual kinesin-5 Cin8 motors is modulated by loop 8, ionic strength and microtubule geometry. *EMBO J.* 30:4942–4954.
16. Britto, M., A. Goulet, …, R. A. Cross. 2016. *Schizosaccharomyces pombe* kinesin-5 switches direction using a steric blocking mechanism. *Proc. Natl. Acad. Sci. USA.* 113:E7483–E7489.
17. Hoyt, M. A., L. He, …, W. S. Saunders. 1992. Two *Saccharomyces cerevisiae* kinesin-related gene products required for mitotic spindle assembly. *J. Cell Biol.* 118:109–120.
18. Roof, D. M., P. B. Meluh, and M. D. Rose. 1992. Kinesin-related proteins required for assembly of the mitotic spindle. *J. Cell Biol.* 118:95–108.
19. Straight, A. F., J. W. Sedat, and A. W. Murray. 1998. Time-lapse microscopy reveals unique roles for kinesins during anaphase in budding yeast. *J. Cell Biol.* 143:687–694.
20. Saito, N., and K. Kaneko. 2017. Embedding dual function into molecular motors through collective motion. *Sci. Rep.* 7:44288.
21. Düselder, A., V. Fridman, …, C. F. Schmidt. 2015. Deletion of the tail domain of the kinesin-5 Cin8 affects its directionality. *J. Biol. Chem.* 290:16841–16850.
22. Shapira, O., and L. Gheber. 2016. Motile properties of the bi-directional kinesin-5 Cin8 are affected by phosphorylation in its motor domain. *Sci. Rep.* 6:25597.
23. Shapira, O., A. Goldstein, …, L. Gheber. 2017. Possible physiological role for bi-directional motility and motor clustering of the mitotic kinesin-5 Cin8. *J. Cell Sci.* 130:725–734.
24. Blackwell, R., C. Edelmaier, …, M. D. Betterton. 2017. Physical determinants of bipolar mitotic spindle assembly and stability in fission yeast. *Sci. Adv.* 3:e1601603.
25. Korneev, M. J., S. Lakämper, and C. F. Schmidt. 2007. Load-dependent release limits the processive stepping of the tetrameric Eg5 motor. *Eur. Biophys. J.* 36:675–681.
26. Shimamoto, Y., S. Forth, and T. M. Kapoor. 2015. Measuring pushing and braking forces generated by ensembles of Kinesin-5 crosslinking two microtubules. *Dev. Cell.* 34:669–681.
27. Block, S. M., L. S. B. Goldstein, and B. J. Schnapp. 1990. Bead movement by single kinesin molecules studied with optical tweezers. *Nature.* 348:348–352.
28. Carter, N. J., and R. A. Cross. 2005. Mechanics of the kinesin step. *Nature.* 435:308–312.
29. Valentine, M. T., P. M. Fordyce, …, S. M. Block. 2006. Individual dimers of the mitotic kinesin motor Eg5 step processively and support substantial loads in vitro. *Nat. Cell Biol.* 8:470–476.
30. Coy, D. L., M. Wagenbach, and J. Howard. 1999. Kinesin takes one 8-nm step for each ATP that it hydrolyzes. *J. Biol. Chem.* 274:3667–3671.
31. Fallesen, T. L., J. C. Macosko, and G. Holzwarth. 2011. Measuring the number and spacing of molecular motors propelling a gliding microtubule. *Phys. Rev. E Stat. Nonlin. Soft Matter Phys.* 83:011918.
32. Castoldi, M., and A. V. Popov. 2003. Purification of brain tubulin through two cycles of polymerization-depolymerization in a high-molarity buffer. *Protein Expr. Purif.* 32:83–88.
33. Hyman, A., D. Drechsel, …, T. Mitchison. 1991. Preparation of modified tubulins. *Methods Enzymol.* 196:478–485.
34. Fallesen, T. L., J. C. Macosko, and G. Holzwarth. 2011. Force-velocity relationship for multiple kinesin motors pulling a magnetic bead. *Eur. Biophys. J.* 40:1071–1079.
35. Phelps, K. K., and R. A. Walker. 2000. NEM tubulin inhibits microtubule minus end assembly by a reversible capping mechanism. *Biochemistry.* 39:3877–3885.

36. Duke, T., T. E. Holy, and S. Leibler. 1995. Gliding assays for motor proteins: a theoretical analysis. *Phys. Rev. Lett.* 74:330–334.

37. Hunt, A. J., F. Gittes, and J. Howard. 1994. The force exerted by a single kinesin molecule against a viscous load. *Biophys. J.* 67:766–781.

38. Nicholas, M. P., L. Rao, and A. Gennerich. 2014. Covalent immobilization of microtubules on glass surfaces for molecular motor force measurements and other single-molecule assays. *Methods Mol. Biol.* 1136:137–169.

39. Lansky, Z., M. Braun, …, S. Diez. 2015. Diffusible crosslinkers generate directed forces in microtubule networks. *Cell.* 160:1159–1168.

40. Berg-Sørensen, K., and H. Flyvbjerg. 2004. Power spectrum analysis for optical tweezers. *Rev. Sci. Instrum.* 75:594.

41. Anderson, T. 1971. The Statistical Analysis of Time Series. John Wiley & Sons, New York.

42. Berg, B. A. 1992. Double jackknife bias-corrected estimators. *Comput. Phys. Commun.* 69:7–14.

43. Furuta, K., A. Furuta, …, H. Kojima. 2013. Measuring collective transport by defined numbers of processive and nonprocessive kinesin motors. *Proc. Natl. Acad. Sci. USA.* 110:501–506.

44. Jamison, D. K., J. W. Driver, and M. R. Diehl. 2012. Cooperative responses of multiple kinesins to variable and constant loads. *J. Biol. Chem.* 287:3357–3365.

45. Visscher, K., M. J. Schnitzer, and S. M. Block. 1999. Single kinesin molecules studied with a molecular force clamp. *Nature.* 400:184–189.

46. Leduc, C., F. Ruhnow, …, S. Diez. 2007. Detection of fractional steps in cargo movement by the collective operation of kinesin-1 motors. *Proc. Natl. Acad. Sci. USA.* 104:10847–10852.

47. Avunie-Masala, R., N. Movshovich, …, L. Gheber. 2011. Phospho-regulation of kinesin-5 during anaphase spindle elongation. *J. Cell Sci.* 124:873–878.

48. Fink, G., L. Hajdo, …, S. Diez. 2009. The mitotic kinesin-14 Ncd drives directional microtubule-microtubule sliding. *Nat. Cell Biol.* 11:717–723.

49. Popchock, A. R., K. F. Tseng, …, W. Qiu. 2017. The mitotic kinesin-14 KlpA contains a context-dependent directionality switch. *Nat. Commun.* 8:13999.

Biophysical Journal, Volume *113*

Supplemental Information

Ensembles of Bidirectional Kinesin Cin8 Produce Additive Forces in Both Directions of Movement

Todd Fallesen, Johanna Roostalu, Christian Duellberg, Gunnar Pruessner, and Thomas Surrey

SUPPLEMENTAL TABLES AND FIGURES

Motor	% (-) Directed	% Not Moving	% (+) Directed	Total MTs
Kinesin-1	2.2	0.5	97.3	184.0
Cin8 5nM	53.0	16.7	30.3	66.0
Cin8 10nM	55.3	34.0	12.8	47.0
Cin8 50nM	0.0	13.6	86.4	110.0
Eg5	5.8	0.0	94.2	103.0

Table S1: Proportion of microtubules in plus and minus directions.
Total MTs refers to total number of microtubules observed for this calculation.

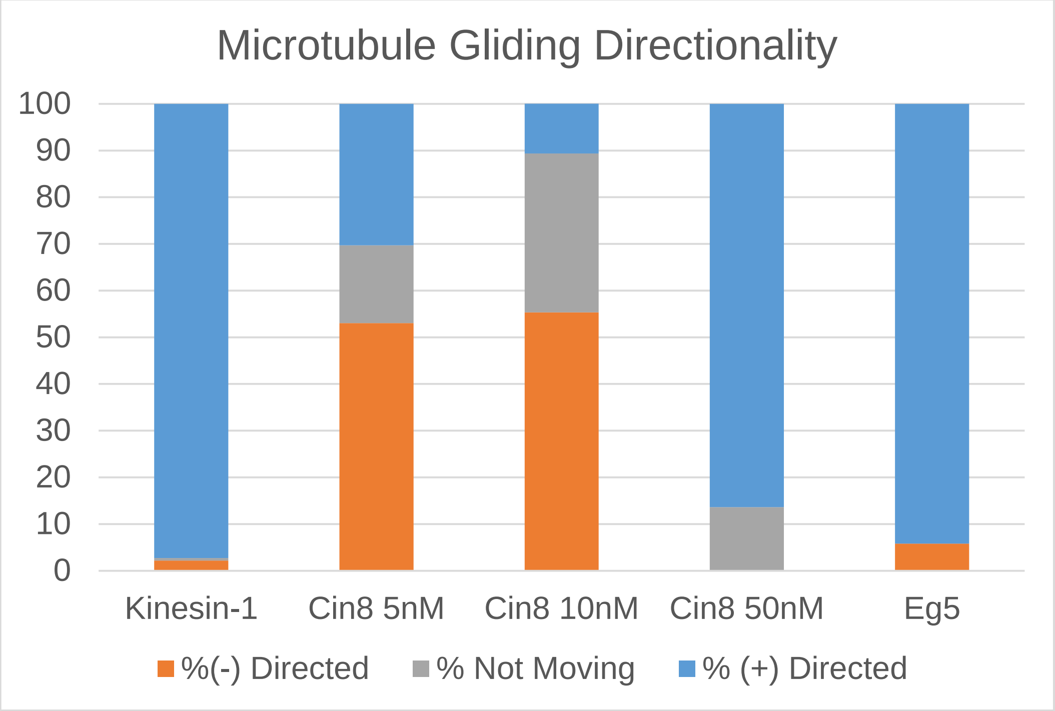

Figure S1. Microtubule polarity marking.
Gliding direction of polarity marked microtubules was observed for 250 nM kinesin-1, 5, 10 and 50 nM Cin8, and 200 nM Eg5. Approximately 98% of the microtubules gliding on kinesin-1 showed the bright polarity mark lagging, as expected for plus end directed motion. Therefore, only ~ 2% of the microtubules with a single polarity mark were incorrectly labelled.

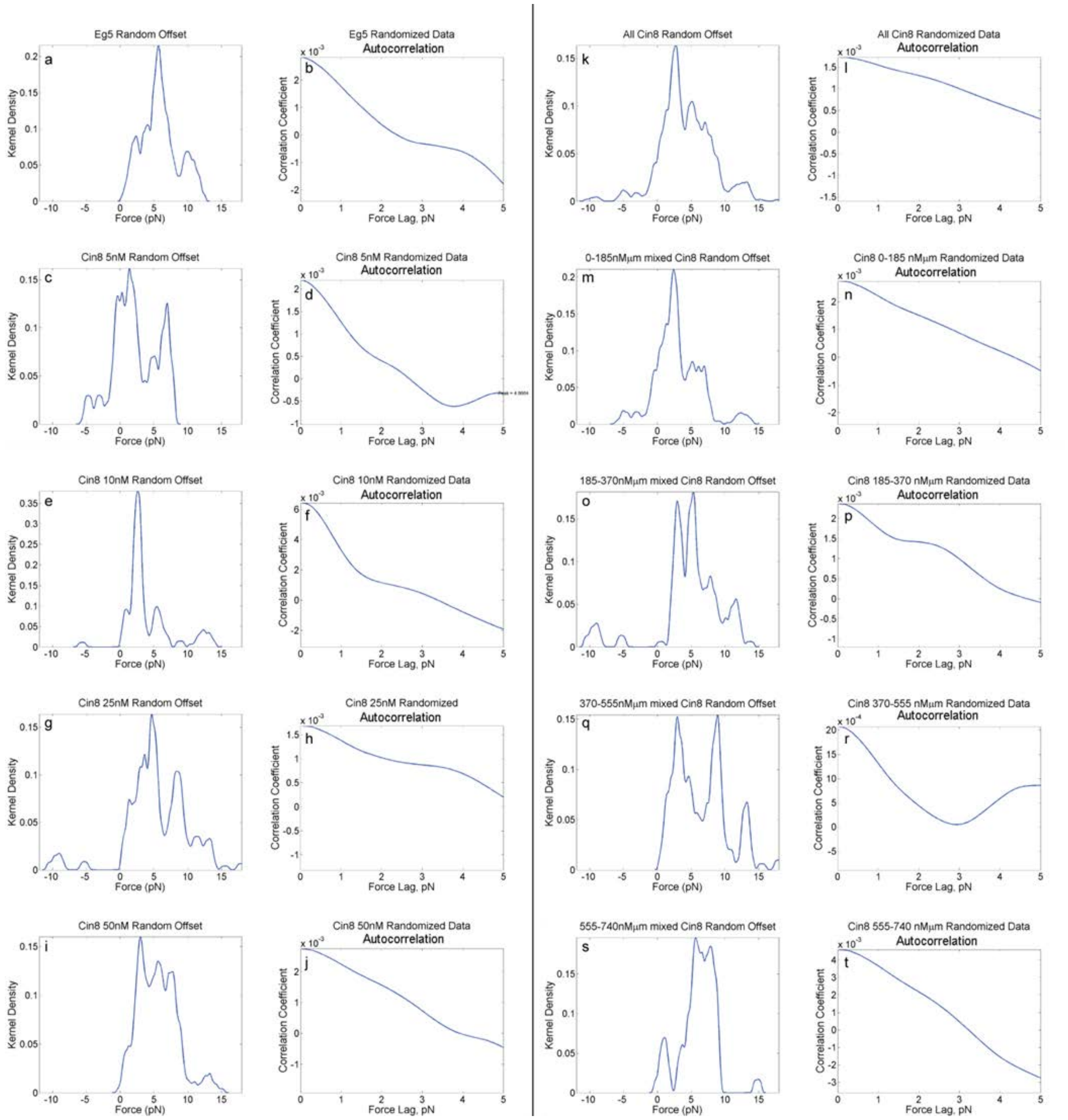

Figure S2. Analysis controls using randomized data

For control purposes, each data set was reanalyzed by kernel density estimation after adding a random value between 0-1.3 pN to each data point. No well-defined structure was seen in kernel density estimation after data randomization, and peaks were undefined in autocorrelations. Kernel density estimations (a, c, e, g, i, k, m, o, q, s) and the corresponding autocorrelation functions (b, d, f, h, j, l, n, p, r, t) are shown for the randomized data sets as indicated. Data presented in (k), (l) is for all Cin8 pause analysis data aggregated together.

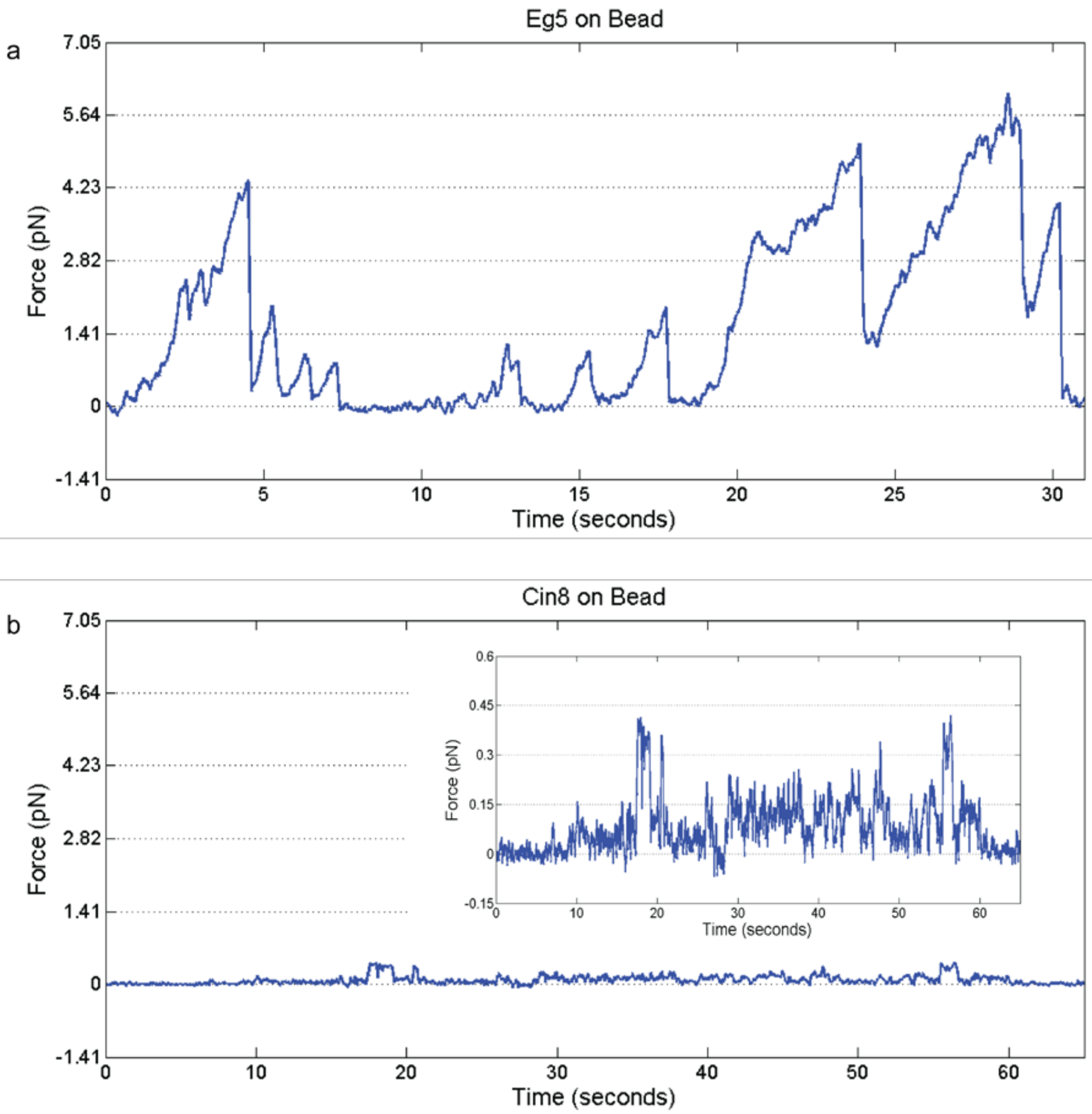

Figure S3. Eg5 and Cin8 on bead control

(a) Force generated against optical trap by Eg5-GFP molecules bound to beads, after beads are placed on surface immobilized microtubules. Trap stiffness is 0.061 pN/nm. Force generated is consistent with 1-4 molecules of Eg5. Eg5-GFP was bound to beads by anti-GFP antibodies. Dashed lines are spaced at 1.41 pN, the value found in the correlation in Figure 1e. ***(b)*** Force generated against optical trap by Cin8-GFP molecules bound via anti-GFP antibodies to beads, after beads are placed on microtubules fixed to surface. Trap Stiffness is 0.0061 pN/nm. Dashed lines are at 1.41 pN for comparison. Lack of force generation could be due to an intrinsic motile property of Cin8 through due to its bidirectionality, or may indicate that Cin8 is not compatible with bead attachment and optical trapping. ***(inset)*** Zoomed view of Cin8-GFP-microtubule interaction.

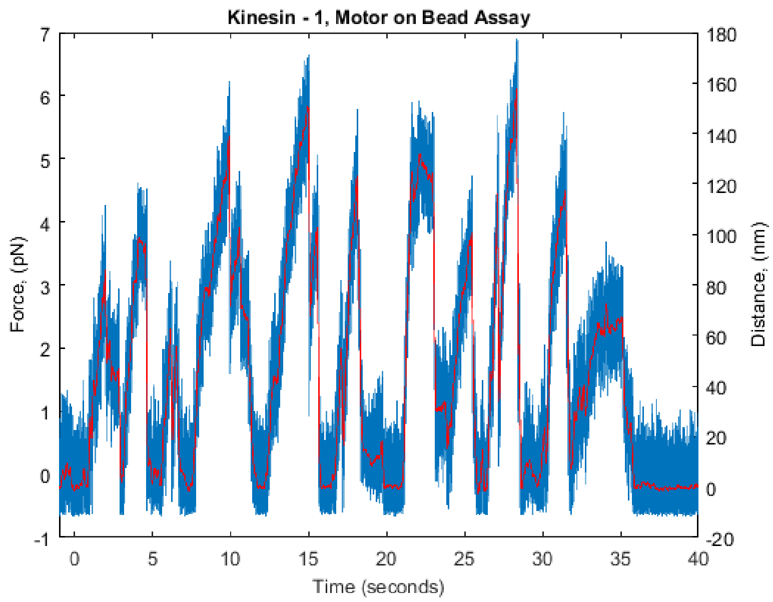

Figure S4. Kinesin-1 on bead control

Force generated by kinesin-1 molecules adsorbed to a caseine-coated carboxylated bead as the kinesin-1 walks along an immoblized microtubule transporting the bead out of the center of the optical trap. Trap stiffness was 0.0379 pN/nm.

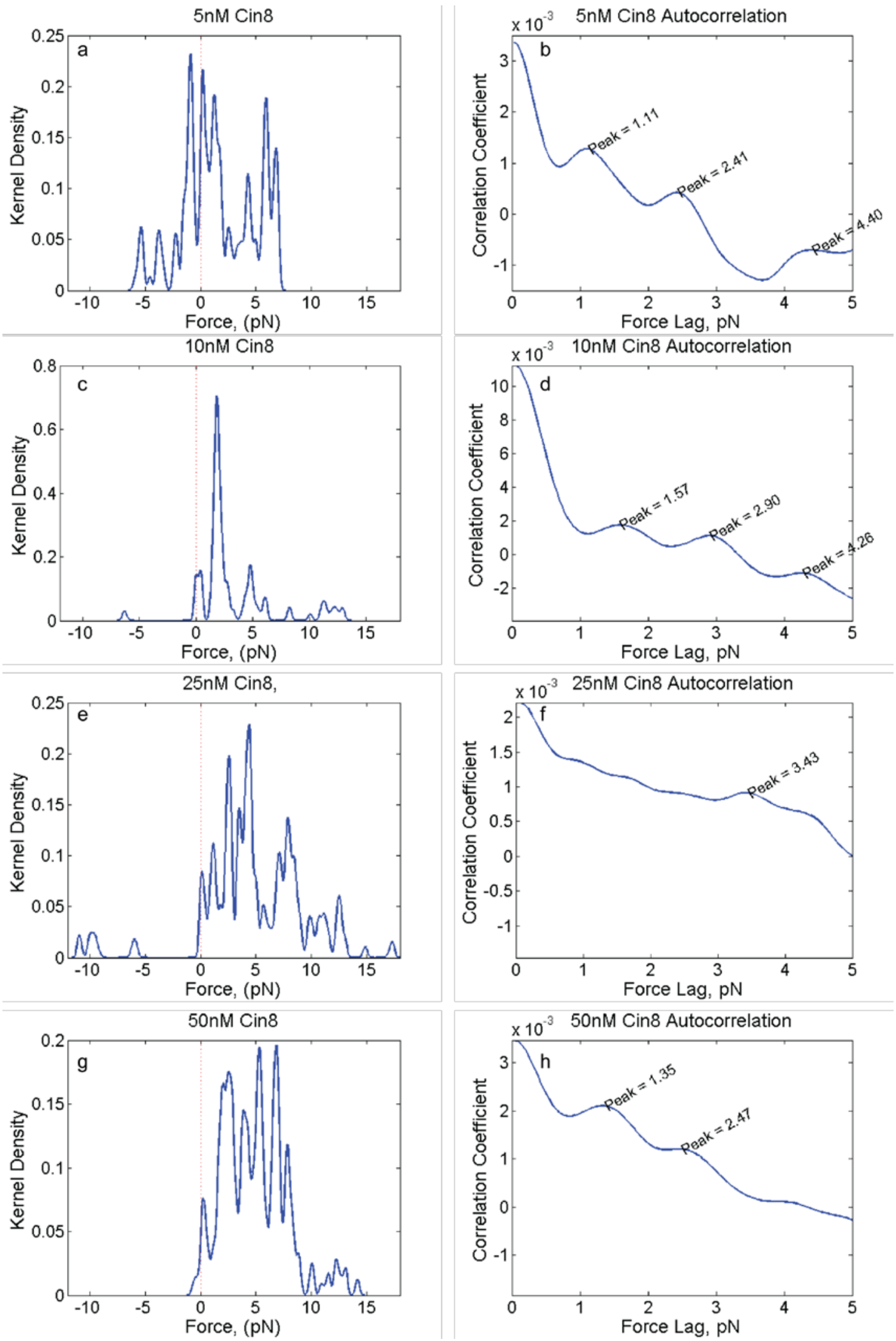

Figure S5. Autocorrelation analysis of Cin8 pause forces measured at different Cin8 concentrations

Kernel density estimation of Cin8 pause forces for experiments performed at different motor concentrations as indicated in panels a, c, e and g, while the autocorrelation functions for the respective Cin8 motor concentration is shown in panels b, d, f and h.

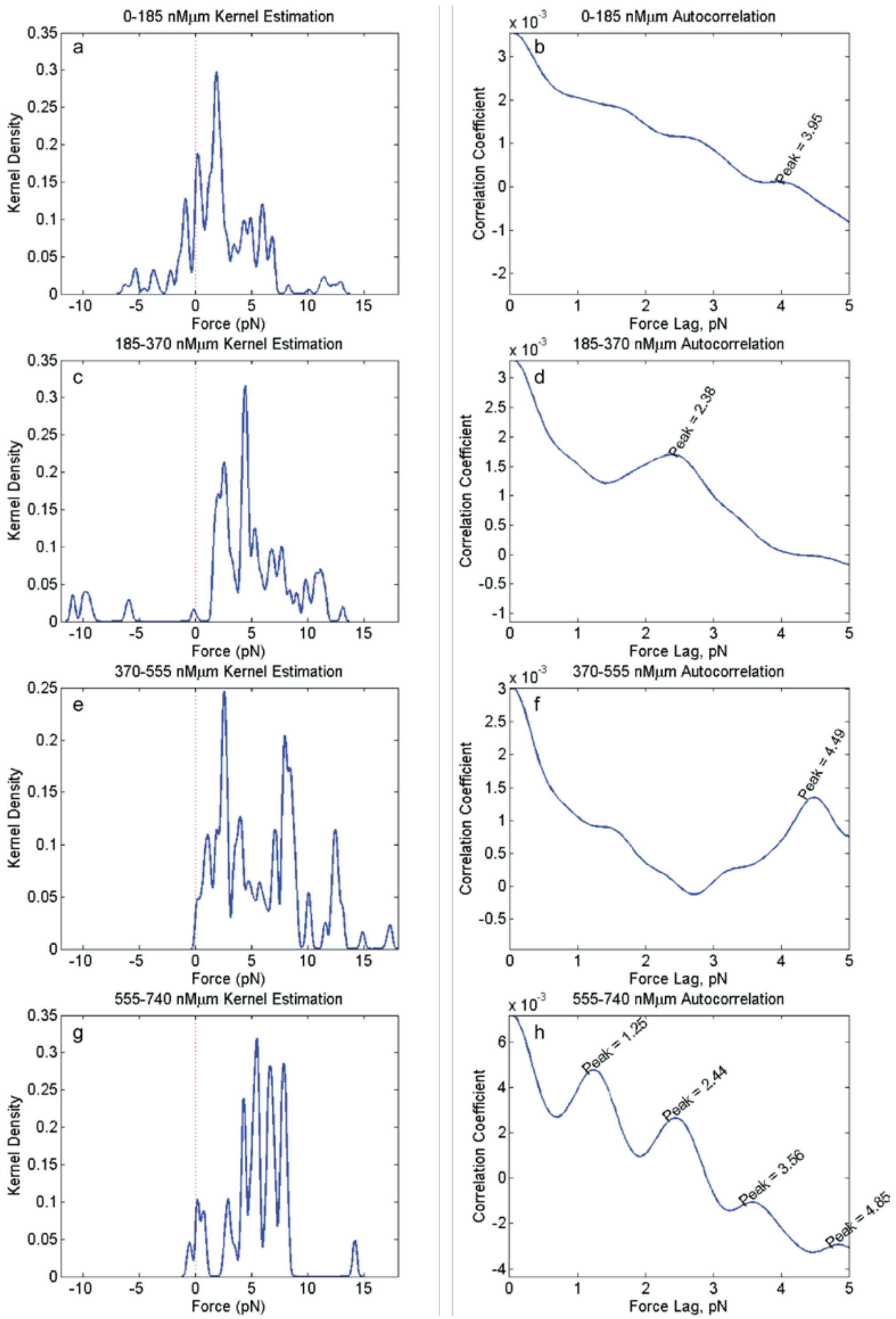

Figure S6. Autocorrelation analysis of Cin8 pause forces after recategorization

Kernel density estimation for all convoluted microtubule length – Cin8 concentration groups are shown in panels *a*, *c*, *e* and *g*. Autocorrelation functions for the respective panels are shown in *b*, *d*, *f* and *h*. Strong correlations are noted at higher nMµm categories, consistent with high motor number.
